# Truncated Dyrk1A aggravates neuronal apoptosis by inhibiting ASF‐mediated Bcl‐x exon 2b inclusion

**DOI:** 10.1111/cns.14493

**Published:** 2023-10-21

**Authors:** Shuqiang Zhang, Junjie Zhong, Lian Xu, Yue Wu, Jie Xu, Jianhua Shi, Zhikai Gu, Xiaoyu Li, Nana Jin

**Affiliations:** ^1^ College of Life Sciences Henan Normal University Xinxiang China; ^2^ Key Laboratory of Neuroregeneration of Jiangsu and Ministry of Education, NMPA Key Laboratory for Research and Evaluation of Tissue Engineering Technology Products, Co‐Innovation Center of Neuroregeneration Nantong University Nantong China; ^3^ Department of Neurosurgery, Institutes of Brain Science, State Key Laboratory for Medical Neurobiology, Fudan University Huashan Hospital Shanghai Medical College‐Fudan University Shanghai China; ^4^ Department of Neurosurgery The Affiliated Hospital of Nantong University Nantong China; ^5^ Institute for translational neuroscience The Second Affiliated Hospital of Nantong University Nantong China

**Keywords:** alternative splicing, apoptosis, ASF, Bcl‐x, Dyrk1A

## Abstract

**Aim:**

Aggravated neuronal loss, caused mainly by neuronal apoptosis, is observed in the brain of patients with Alzheimer's disease (AD) and animal models of AD. A truncated form of Dual‐specific and tyrosine phosphorylation‐regulated protein kinase 1A (Dyrk1A) plays a vital role in AD pathogenesis. Downregulation of anti‐apoptotic Bcl‐xL is tightly correlated with neuronal loss in AD. However, the molecular regulation of neuronal apoptosis and Bcl‐x expression by Dyrk1A in AD remains largely elusive. Here, we aimed to explore the role and molecular mechanism of Dyrk1A in apoptosis.

**Methods:**

Cell Counting Kit‐8 (CCK8), flow cytometry, and TdT‐mediated dUTP Nick‐End Labeling (TUNEL) were used to check apoptosis. The cells, transfected with Dyrk1A or/and ASF with Bcl‐x minigene, were used to assay Bcl‐x expression by RT‐PCR and Western blots. Co‐immunoprecipitation, autoradiography, and immunofluorescence were conducted to check the interaction of ASF and Dyrk1A. Gene set enrichment analysis (GSEA) of apoptosis‐related genes was performed in mice overexpressing Dyrk1A (TgDyrk1A) and AD model 5xFAD mice.

**Results:**

Dyrk1A promoted Bcl‐xS expression and apoptosis. Splicing factor ASF promoted Bcl‐x exon 2b inclusion, leading to increased Bcl‐xL expression. Dyrk1A suppressed ASF‐mediated Bcl‐x exon 2b inclusion via phosphorylation. The C‐terminus deletion of Dyrk1A facilitated its binding and kinase activity to ASF. Moreover, Dyrk1a_1–483_ further suppressed the ASF‐mediated Bcl‐x exon 2b inclusion and aggravated apoptosis. The truncated Dyrk1A, increased Bcl‐xS, and enrichment of apoptosis‐related genes was observed in the brain of 5xFAD mice.

**Conclusions:**

We speculate that increased Dyrk1A and truncated Dyrk1A may aggravate neuronal apoptosis by decreasing the ratio of Bcl‐xL/Bcl‐xS via phosphorylating ASF in AD.

## Graphical abstract

The full length Dyrk1A phosphorylated ASF and inhibited ASF‐mediated Bcl‐xL exon 2b inclusion leading to cell apoptosis. Moreover, the truncated Dyrk1A deleting the C‐terminus exhibited equidistribution in the nucleoplasm and stronger interaction with ASF, leading to decreased ratio of Bcl‐xL/Bcl‐xS and neuronal apoptosis in AD.

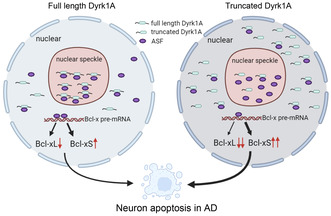

## 1 INTRODUCTION

Alzheimer's disease (AD) is the most common form of dementia and is characterized by three hallmark histopathologic features: the extracellular amyloid‐β (Aβ) plaques, intracellular neurofibrillary tangles, and considerable neuron loss.^1^, ^2^, ^3^ The aggravated loss of brain mass and neuron‐are pathological features detected even in patients with mild AD.^4^ The positive correlation between Braak's stages and neuronal loss is observed in the hippocampus and frontal cortex.^5^ Apoptotic and non‐apoptotic forms of death (pyroptosis, necrosis, necroptosis, and ferroptosis) contribute to the neuronal loss.^6^ Apoptotic neuronal death is commonly observed in the brains of patients with AD,^7^, ^8^ AD mice models,^9^ hiPSC neuronal culture,^10^ and primary neuronal culture.^11^ Thus, the investigation of the molecular mechanism of neuronal apoptosis could be conducive to the development of a novel therapeutic approach in AD.

Bcl‐x, encoded by *the Bcl‐2‐like 1* gene (*Bcl2l1*), is a member of the Bcl‐2 family. An alternative selection of the 5′‐splice site in its exon 2b leads to the alternative splicing of Bcl‐x exon 2b and generates a long isoform of Bcl‐x (Bcl‐xL) and a short isoform (Bcl‐xS).^12^ They perform opposing roles in apoptosis: Bcl‐xL acts as an apoptotic inhibitor, while Bcl‐xS acts as an apoptotic activator.^12^ Bcl‐xL is the major Bcl‐x isoform in the central nervous system (CNS) and is broadly found in the embryonic and postnatal brain.^13^, ^14^ The conditional knockout of Bcl‐xL in the neuronal progenitor cells of mice results in increased neuronal death in the brain and neurobehavioral deficits.^15^ The neuronal apoptosis induced by Aβ is characterized by the downregulation of Bcl‐2, Bcl‐W, and Bcl‐xL.^16^, ^17^ Overexpression of Bcl‐xL in neurons diminishes brain infarction after cerebral ischemia^18^ and the CNS neurodegeneration after lesion.^19^ Thus, the regulation of Bcl‐xL expression might be a potential therapeutic target for impaired neuronal development and neurodegeneration.

Dual‐specific and tyrosine phosphorylation‐regulated protein kinase 1A (Dyrk1A), a proline‐ and arginine‐directed Ser/Thr kinase, is abundantly expressed in the brain and plays important roles in cell proliferation, survival, neurogenesis, neuronal death, and development of the central nervous system. It is overexpressed in Down syndrome (DS) as a result of trisomy 21.^20^ Dyrk1A transgenic mice (TgDyrk1A) exhibit DS‐like mental retardation. Dyrk1A is also a risk gene for patients with DS to develop precocious neurodegeneration of the Alzheimer‐type.^21^ Dyrk1A can directly phosphorylate Amyloid precursor protein (APP) at Thr668, and presenilin 1(PSEN1) at Thr354. In the AD brain, Dyrk1A is truncated at the C‐terminus by calpain I, which enhances its kinase activity, resulting in the tau hyperphosphorylation and dysregulation of tau exon 10 contributing to early tau pathology.^22^

Alternative splicing is controlled by exonic and intronic enhancers and silencers, which in turn are regulated by splicing factors. Dyrk1A phosphorylates several splicing factors, including SF3b1/SAP155, cyclin L2, ASF, 9G8, SC35, and SRp55.^23^, ^24^, ^25^, ^26^, ^27^, ^28^ Engineering Dyrk1A overdosage yields DS‐characteristic cortical splicing aberrations.^29^ The reduced expression of Bcl‐xL is observed in the hippocampus of DS model Ts65Dn mice.^30^ In the present study, we explored the role and molecular mechanism of Dyrk1A in apoptosis. Our results highlight a potential mechanism for the increased neuronal apoptosis and neuronal loss in AD.

## 2 MATERIALS AND METHODS

### 2.1 Animals

5xFAD hemizygous (B6.Cg‐Tg(APPSwFlLon, PSEN1*M146L*L286V) 6799Vas/Mmjax, Stock number 34848‐JAX) and its wild‐type control mice were obtained from the Jackson Laboratory. Mice were housed (five to six animals per cage) with a 12 h/12 h light/dark cycle and with ad libitum access to food and water. The experimental protocols were approved by the Ethics Committees of Nantong University (Approval ID: S20210303‐013).

### 2.2 Plasmids, proteins, and antibodies

pCI/Dyrk1A and its deletion mutants were constructed and confirmed by Sanger sequencing. pCI/Dyrk1As were tagged with FLAG at the C‐terminus. pCEP4/ASF‐HA, pCEP4/SC35‐HA, and pCEP4/SRp55‐HA were kind gifts from Dr. Tarn of the Institute of Biomedical Sciences, Academia Sinica, Taiwan. The Bcl‐x mini‐gene, comprising Bcl‐x exons 2 and 3 and intron 2 was a gift from Dr. Jianhua Zhou of the University of Massachusetts Medical School. pCEP4/ASF_S3A_‐HA, ASF was mutated at Ser‐227, Ser‐234, and Ser‐238 to three Ala, as described previously.^25^ Small inference RNAs (siRNAs) of Dyrk1A and ASF, and Monoclonal anti‐Bcl‐xL were obtained from Santa Cruz Biotechnology. Monoclonal antibody 8D9 was raised against a histidine‐tagged protein containing the first 160 residues of rat Dyrk1A.^31^ The monoclonal anti‐HA, polyclonal anti‐FLAG, and monoclonal anti‐Dyrk1A (N‐terminal) were purchased from Sigma. Polyclonal Bcl‐xS, and monoclonal anti‐NeuN were purchased from Thermo Fisher Scientific. Horseradish peroxidase (HRP) conjugated secondary antibodies were obtained from Jackson ImmunoResearch Laboratories (West Grove, PA). Enhanced chemiluminescence ECL kit was bought from Thermo Fisher Scientific.

### 2.3 Cell culture and transfection

HEK‐293A, HEK‐293FT, Hela, and U87 cells were maintained in Dulbecco's modified Eagle's medium (DMEM) supplemented with 10% fetal bovine serum (FBS, Sigma) at 37°C. All transfections were performed with Lipofectamine 3000 (Invitrogen) according to the manufacture's protocols. For FBS starvation treatment, the cells were maintained in DMEM without FBS for 24 h after transfection.

### 2.4 RNA Interference

For inhibition of ASF and Dyrk1A expression, HEK‐293 cells cultured in 24‐well plates were transfected with various amounts of short interfering RNA (siRNA) using Lipofectamine 3000. After a 48 h transfection, cells were lysed, and protein and RNA were extracted as described above. siRNA target sequences to ASF were 5′‐GTAGAACCCATGTTGTATA‐3′, 5′‐GTTCCAATGTATTGGTGTA‐3′, and 5′‐GGAGCTGGATCATTGGATT‐3′ (Santa Cruz Biotechnology). Dyrk1A SMARTpool target sequences to Dyrk1A were 5′‐TAAGGATGCTTGATTATGA‐3′, 5′‐GCTAATACCTTGGACTTTG‐3′, 5′‐GAAAACAGCTGATGAAGGT‐3′, and 5′‐AAACTCGAATTCAACCTTA‐3′ (Dharmacon, Lafayette, CO). Both strands of siRNAs had two uridines at 3′ end. The same concentration of scrambled siRNA was used for controls.

### 2.5 Western blot analysis

Cultured cells were lysed with Laemmli buffer and boiled for 5 min, and the protein concentration was measured by A660 (Thermo Fisher Scientific). Equal concentration of protein from each sample was separated by sodium dodecyl sulfate (SDS)–polyacrylamide gel electrophoresis (PAGE) and electro‐blotted onto a PVDF membrane. After blocking with 5% fat‐free milk, the membrane was incubated with primary antibodies overnight at room temperature in the presence of 0.1% NaN3 in 5% fat‐free milk. After washing with TBST (Tris–HCl, Ph 7.4, 150 Mm NaCl, 0.05% Tween 20) three times, the membrane was incubated with the corresponding HRP‐conjugated secondary antibody for ~2 h. After washing with TBST, the blot was visualized by enhanced chemiluminescence and quantified by densitometry using Multi Gauge V2.3 software (Fuji Film). The full unedited blot for all blots were showed in Data S1.

### 2.6 Total RNA extraction and RT‐PCR analysis

Total RNA was extracted from cultured cells using Rneasy mini kit (Qiagen, GmbH) according to the manufacturer's protocol. One microgram total RNA was reverse transcribed using Omniscript Reverse Transcription Kit (Qiagen, GmbH) following the manufacturer's protocol. PCR was performed using Prime‐START HS DNA Polymerase (Takara Bio Inc., Otsu) with primers (forward 5′‐ACGACTCACTATAGGCTAG‐3′ and reverse 5′‐ATTGTTCCCATAGAGTTCCAC‐3′ for exogenous; forward 5′‐CAGGGACAGCATATCAGAGC‐3′ and reverse 5′‐TTCCGACTGAAGAGTGAG‐3′ for endogenous) to measure alternative splicing of Bcl‐x exon 2b under conditions: at 98°C for 3 min, at 98°C for 10 s, and at 68°C for 40 s for 30 cycles, and then at 68°C for 10 min for extension. The PCR products were resolved on 1.2% agarose gels and quantitated using the Molecular Imager system (Bio‐Rad).

### 2.7 Phosphorylation of ASF by Dyrk1A in cultured cells

HEK‐293 cells were co‐transfected with pCEP4/ASF‐HA or pCEP4/ASF_3A_‐HA with/without pCI/Dyrk1A or its deletion mutants and cultured in DMEM supplemented with 10% fetal bovine serum. At 45 h post‐transfection, the medium was replaced with [^32^P]orthophosphate (10 mCi) in DMEM (−phosphate) with 10% FBS. After a 3‐h incubation, the cells were harvested in lysis buffer (50 mM Tris–HCl, Ph 7.4, 150 mM NaCl, 50 mM NaF,1 mM Na_3_VO_4_, 50 Mm okadaic acid, 0.1% Triton X‐100, 0.1% Nonidet P‐40, 0.25% sodium deoxycholate, 2 mM EDTA, 1 mM phenylmethylsulfonyl fluoride, and 10 mg/mL aprotinin, leupeptin, and pepstatin). Insoluble materials were removed by centrifugation, and the supernatant was incubated with anti‐ASF (for endogenous ASF) or anti‐HA (for overexpressed ASF) pre‐coupled protein G beads overnight. After washing with lysis buffer and TBS, immunoprecipitated ASF was analyzed by autoradiography following SDS‐PAGE.

### 2.8 Co‐immunoprecipitation

The HEK‐293FT cells were seeded in 12‐well plates and transfected with pCI/Dyrk1A and its deletion mutants on the second day and left undisturbed for 48 h. The cells were washed with ice‐cold PBS three times and lysed with lysis buffer (50 mM Tris–HCl, Ph 7.4, 150 mM NaCl, 50 mM NaF,1 mM Na_3_VO_4_, 0.25% sodium deoxychalate, 2 mM EDTA, 1 mM phenylmethylsulfonyl fluoride, and 10 mg/mL aprotinin, leupeptin, and pepstatin). The cell lysates were sonicated 20 times with pulses 0.5 s on and 2 s off at 20% power. The insoluble fractions were removed by centrifugation at 15,000 × *g*, for 10 min at 4°C. Monoclonal ASF antibody coupled protein G‐agarose beads (ThermoFisher Scientific) were incubated with the cell supernatant overnight at 4°C. The beads were washed four times with TBS. The Laemmli sample buffer was added to the beads and boiled to elute the bound proteins from the beads. The immunoprecipitated and co‐immunoprecipitated proteins were analyzed by western blotting using corresponding antibodies.

### 2.9 Apoptosis determination

Cultured cells were seeded in 12‐well plates at a density of 2 × 10^5^ cells/well and transfected with the indicated plasmids for 46 h. The cells were treated with 2 mM and 6 mM H_2_O_2_ for 2 h. After H_2_O_2_ treatment, all suspended and adherent cells were collected, stained with annexin V–PI double staining kit Flow Cytometry Kit for Apoptosis (Sigma), and subjected to flow cytometry.

### 2.10 Cell viability assay

Cells were seeded in 96‐well plates at a density of 5 × 10^3^ cells/well with triplications, and cultured at 37°C for 24 h. The cells were transfected according to the requirement of corresponding groups and further cultured for 48 h. Then, 10 μL CCK‐8 (Dojindo) was added to each well, and the cells were further incubated for 1 h. The optical density (OD) values were measured at 450 nm using a plate reader Synergy4 (Biotek).

### 2.11 TUNEL assay

The DeadEnd™ Fluorometric TUNEL System (Sigma) was used to detect apoptosis. The cells transfected with the above plasmids were incubated with TUNEL reaction mixture at 37°C for 1 h in a humid atmosphere. 4′, 6‐diamidino‐2‐phenylindole (DAPI) staining solution was used to label the nuclei. The cells with nuclei intensely labeled by green fluorescence were identified as TUNEL‐positive apoptotic cells. Images were captured on a TCS‐SP2 confocal microscope (Leica).

### 2.12 Immunofluorescence staining

HeLa cells were plated on glass coverslips in 24‐well plates. Two days post‐transfection, the cells were washed with PBS and fixed in 4% paraformaldehyde in PBS for 20 min at room temperature. The cells were permeabilized using 0.5% Triton X‐100 in PBS. After blocking with 10% heat‐inactivated goat serum in PBS containing 0.05% Triton X‐100 for 1 h at 37°C, the cells were incubated with monoclonal HA antibody (1:2000) or/and polyclonal FLAG antibody (1:500) overnight at 4°C. After washing, the cells were incubated with secondary antibodies and TO‐PRO‐3 at room temperature, and the mounted with SlowFade® Gold Antifade Mountant (Invitrogen), and imaged with a TCS‐SP2 confocal microscope (Leica).

### 2.13 Gene set enrichment analysis (GESA)

The expression profiles of hippocampus in the Dyrk1A overexpression mice (TgDyrk1A) (GSE149464) or hippocampus and cortex in the AD model 5xFAD mice (GSE168137) were retrieved from the NCBI GEO database. Gene set enrichment analysis of apoptosis‐related genes was performed using GSEA.^32^ Terms with FDR q‐value ≤0.25 and/or NOM *p*‐value ≤0.05 were defined as a significant enrichment.

### 2.14 Statistical analysis

When appropriate, the data were presented as the means ± standard deviation (SD). The distribution of data in each set of experiments was tested for normality using the D'Agostino– Pearson omnibus test or Shapiro–Wilk test. Data points were analyzed by unpaired two‐tailed Student *t* test for comparisons between two groups, and one‐way ANOVA followed by Tukey's post hoc test and two‐way ANOVA followed by Sidak's multiple comparisons test for comparison among multiple groups using Graphpad Prism v.8.0. The calculated *p*‐values were indicated in the figures.

## 3 RESULTS

### 3.1 Overexpression of Dyrk1A promotes cell apoptosis by decreasing the ratio of Bcl‐xL/Bcl‐xS

**FIGURE 1:**
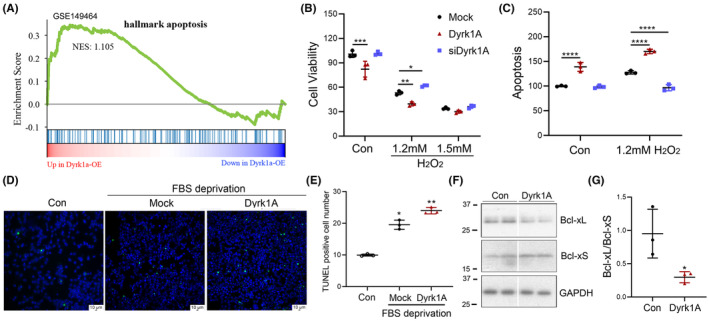
Dyrk1A promotes cell apoptosis and increases expression of pro‐apoptotic protein Bcl‐xS. (A) GESA analysis of the apoptosis gene enrichment of hippocampal RNA sequencing data from Dyrk1A overexpressed (TgDyrk1A) mice at 16 weeks. (B,C) Dyrk1A was overexpressed or silenced in U87 cells. The cells were treated with H_2_O_2_ at the indicated concentration for 2 h. Cell viability was measured by CCK8 (B) and flow cytometry (C). (D,E) HEK‐293 T cells were transfected with Dyrk1A and were deprived of FBS for 24 h. The apoptosis was determined by TUNEL staining (D). The TUNEL positive cells were counted in a total of 1000 cells (E). (F,G) Dyrk1A was overexpressed in HEK‐293A cells for 48 h. The cells were harvested and analyzed by western blot to detect Bcl‐xL, Bcl‐xS, and GAPDH (F). The Bcl‐xL/Bcl‐xS ratio was calculated after densitometry (G). Data are presented as mean ± SD; *n* = 3; *, *p* < 0.05; **, *p* < 0.01.

To investigate the role of Dyrk1A in apoptosis, GSEA analysis of apoptosis‐related genes was performed based on the expression profile of hippocampus in TgDyrk1A mice (GEO149470).^33^ We found that apoptosis‐related genes were slightly enriched in the hippocampus of Dyrk1A overexpressed mice, compared with that of wild‐type mice (NES = 1.105; Figure 1A). To further explore the effect of Dyrk1A on the cell apoptosis, we performed overexpression and knockdown of Dyrk1A in U87 cells (Human glioma cell line) and then treated the cells with 1.2 mM or 1.5 mM H_2_O_2_ for 2 h to induce apoptosis. Overexpression of Dyrk1A reduced cell viability, whereas the knockdown of Dyrk1A did not have any effect (Figure 1B). H_2_O_2_ treatment significantly decreased cell viability in a dose‐dependent manner (Figure 1B). Overexpression of Dyrk1A enhanced, while knockdown of Dyrk1A prevented 1.2 mM H_2_O_2_‐induced cell death (Figure 1B). However, overexpression or knockdown of Dyrk1A showed little effect on 1.5 mM H_2_O_2_‐induced reduction in cell viability (Figure 1B). We also performed flow cytometric analysis of apoptosis following overexpression or silencing of Dyrk1A in U87 cells. Overexpression of Dyrk1A, but not knockdown of Dyrk1A, promoted cell apoptosis (Figure 1C). Overexpression of Dyrk1A exacerbated, whereas knockdown of Dyrk1A attenuated the 1.2 mM H_2_O_2_‐induced apoptosis (Figure 1C). To further confirm the role of Dyrk1A on apoptosis in another cell line, Dyrk1A was overexpressed in HEK‐293 T cells (human embryonic kidney cell) with FBS deprivation to induce apoptosis and the cells were analyzed using TUNEL Assay. Compared with the control group, FBS deprivation drastically increased the number of TUNEL‐positive cells (Figure 1D,E), and overexpression of Dyrk1A further increased this, indicating that Dyrk1A exacerbated the cell apoptosis induced by FBS deprivation (Figure 1D,E). These results suggest that Dyrk1A could promote apoptosis.

**FIGURE 2:**
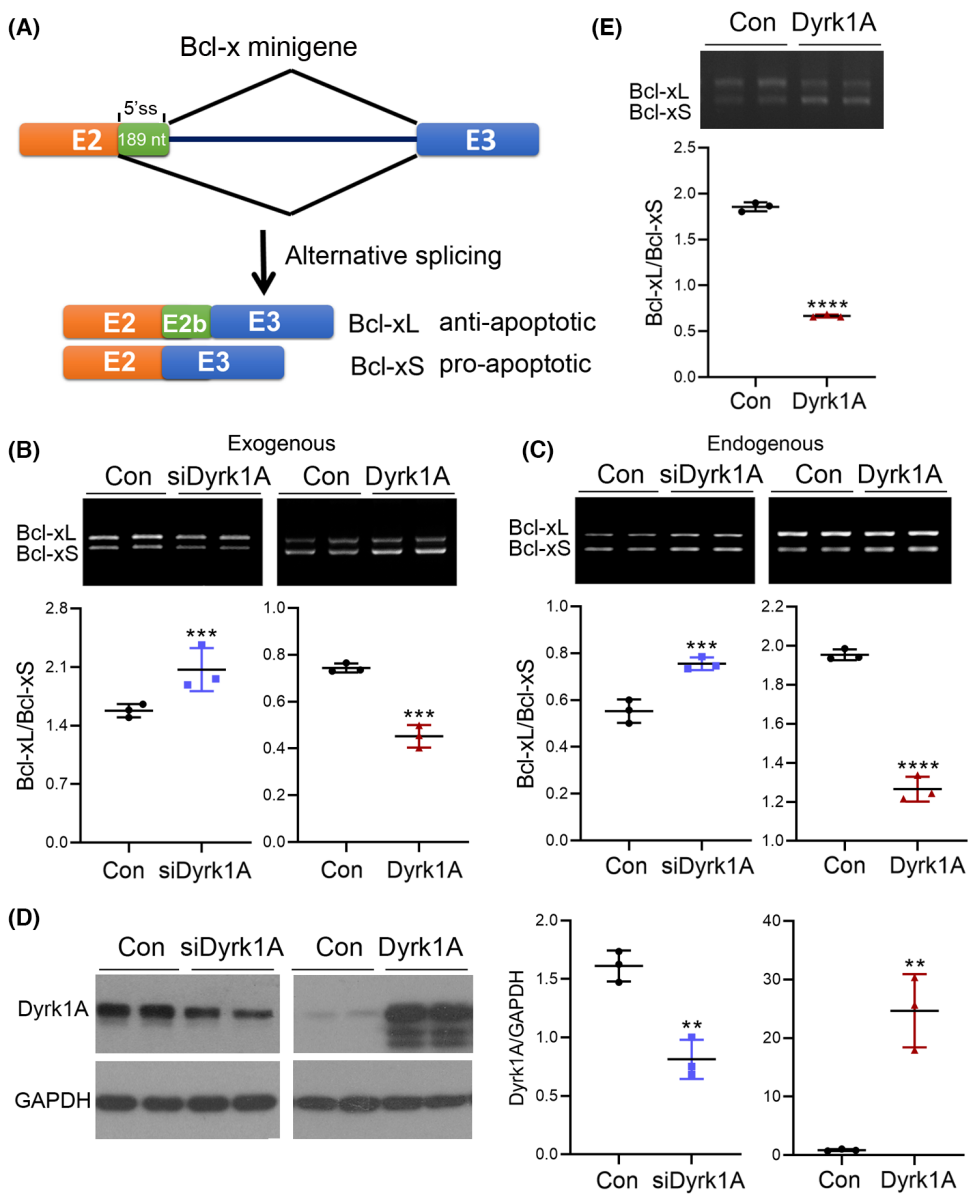
Dyrk1A promotes Bcl‐x exon 2b exclusion leading to enhanced Bcl‐xS expression. (A) A schematic depicting the alternative splicing of Bcl‐x exon 2b. (B–D) siRNA of Dyrk1A or Dyrk1A were co‐transfected with Bcl‐x mini‐gene into HEK‐293A cells. The splicing products of exogenous (B) and endogenous (C) of Bcl‐x exon 2b were analyzed by RT‐PCR using two sets of primers corresponding to endogenous and exogenous Bcl‐x. The Bcl‐xL/Bcl‐xS ratio was calculated following densitometry. The level of Dyrk1A was analyzed by immunoblotting (D). (E) Dyrk1A was co‐transfected with Bcl‐x mini‐gene into U87 cells. Data are presented as mean ± SD; *n* = 3; *, *p* < 0.05; **, *p* < 0.01.

Alternative splicing of exon 2b generates long isoform Bcl‐xL (anti‐apoptotic) and short isoform Bcl‐xS (pro‐apoptotic) with and without exon 2b, respectively (Figure 2A). Our previous study showed that Dyrk1A could regulate the alternative splicing of tau.^25^ Thus, we hypothesized that Dyrk1A may promote apoptosis via regulating alternative splicing of Bcl‐x exon 2b. To this end, we overexpressed Dyrk1A in HEK‐293 T cells and detected the protein level of Bcl‐xL and Bcl‐xS by immunoblotting. Overexpression of Dyrk1A could decrease the protein level of Bcl‐xL while increasing the protein level of Bcl‐xS (Figure 1F). Dyrk1A also significantly decreased the ratio of Bcl‐xL/Bcl‐xS (Figure 1G). These results indicate that Dyrk1A regulated the protein expression of Bcl‐xL and Bcl‐xS and reduced the ratio of Bcl‐xL/Bcl‐xS to promote cell apoptosis.

### 3.2 Dyrk1A promotes Bcl‐x exon 2b exclusion

To study the role of Dyrk1A in the selection of the 5′‐splice site in Bcl‐x exon 2b, we employed Bcl‐x mini‐gene, which consists of exons 2, 2b, and 3 and intron 2 (Figure 2A). Dyrk1A was overexpressed or knocked down by the siRNA in Bcl‐x mini‐gene transfected HEK‐293A cells or normal HEK‐293A cells, and analyzed the splicing products of Bcl‐x exon 2b by RT‐PCR. siDyrk1A successfully knocked down the expression of Dyrk1A (Figure 2D). We found that knockdown of Dyrk1A suppressed exogenous and endogenous Bcl‐x exon 2b exclusion, leading to an increased Bcl‐xL/Bcl‐xS ratio (Figure 2B,C). Conversely, overexpression of Dyrk1A in HEK‐293A cells promoted exogenous and endogenous Bcl‐x exon 2b exclusion, resulting in an increased Bcl‐xS expression (Figure 2B,C). The effect of Dyrk1A on 5′‐splice site selection in exogenous and endogenous Bcl‐x exon 2 showed a similar trend, but a more significant change was observed in the exogenous Bcl‐x exon 2 (Figure 2B,C). Having observed the well‐represented alternative splicing of Bcl‐x exon 2b of Bcl‐x mini‐gene, we then used this mini‐gene for the following experiments. The role of Dyrk1A in the 5′‐splice site selection in U87 cells was also investigated. Similar to that in HEK‐293A cells, overexpression of Dyrk1A promoted the Bcl‐x exon 2b exclusion and enhanced Bcl‐xS expression (Figure 2E) in U87 cells. These results suggest that Dyrk1A promotes Bcl‐x exon 2b exclusion and decreased the Bcl‐xL/Bcl‐xS ratio.

### 3.3 ASF promotes Bcl‐xL expression and enhances cell viability

**FIGURE 3:**
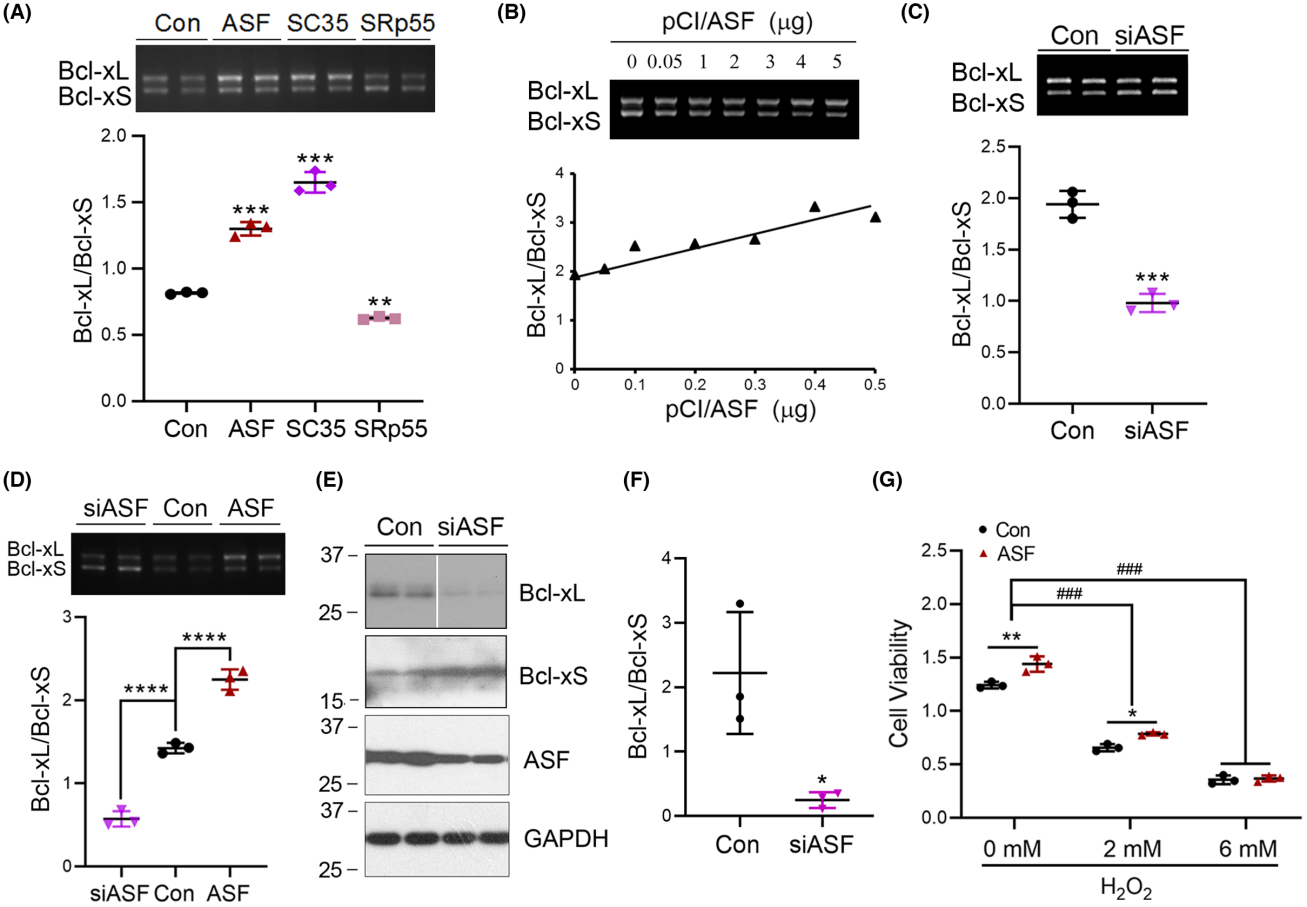
ASF promotes Bcl‐xL expression and increases cell viability. (A) The splicing factors ASF, SC35, and SRp55 were co‐transfected with Bcl‐x mini‐gene into HEK‐293A cells. The splicing products of Bcl‐x exon 2b were analyzed by RT‐PCR. (B,C) ASF was overexpressed or knocked down with siRNA in Bcl‐x mini‐gene transfected HEK‐293A cells. The alternative splicing products of Bcl‐x exon 2b was analyzed by RT‐PCR. The Bcl‐xL/Bcl‐xS ratio was calculated and plotted against the concentration of pCI/ASF (B). (D) U87 cells were co‐transfected with siASF or ASF and Bcl‐x mini‐gene. The splicing products were analyzed by RT‐PCR. The ratio of Bcl‐xL to Bcl‐xS was calculated. (E,F) The expression of ASF was knocked down by siASF. Western blotting was performed to quantify the expression of Bcl‐xL, Bcl‐xS, ASF, and GAPDH. The ratio of Bcl‐xL/Bcl‐xS was calculated (F). (G) The cells were transfected with ASF and treated with different concentration H_2_O_2_ for 2 h. Cell viability was analyzed by CCK8. Data are presented as mean ± SD, *n* = 3; *, *p* < 0.05; **, *p* < 0.01, ***, *p* < 0.001, ****, *p* < 0.0001.

To determine SR proteins that regulate the alternative splicing of Bcl‐x exon 2b, we overexpressed SRs (ASF, SC35, and SRp55) in Bcl‐x mini‐gene‐U87 cells and analyzed the splicing products. We found that ASF and SC35 promoted Bcl‐xL expression, whereas SRp55 enhanced Bcl‐xS expression (Figure 3A). Thus, some SR proteins may act on Bcl‐x exon 2b splicing differentially.

To determine the role of ASF in Bcl‐x exon 2b splicing, we overexpressed ASF in Bcl‐x mini‐gene transfected HEK‐293A cells. We found that ASF increased the ratio of Bcl‐xL/Bcl‐xS in a dose‐dependent manner (Figure 3B). Knockdown of ASF in HEK‐293A cells with siASF promoted Bcl‐xS mRNA (Figure 3C) and protein (Figure 3E) expression and decreased the Bcl‐xL mRNA (Figure 3C) and protein (Figure 3F) expression. Similarly, in U87 cells, the overexpression of ASF promoted Bcl‐xL expression, while knockdown enhanced Bcl‐xS expression (Figure 3D). Overexpression of ASF significantly increased cell viability with or without 2 mM H_2_O_2_ treatment (Figure 3G), not the 6 mM H_2_O_2_. Thus, these results suggest that ASF could enhance cell viability through increasing the expression of Bcl‐xL.

### 3.4 Dyrk1A phosphorylates ASF and suppresses ASF‐mediated Bcl‐x exon 2b inclusion

**FIGURE 4:**
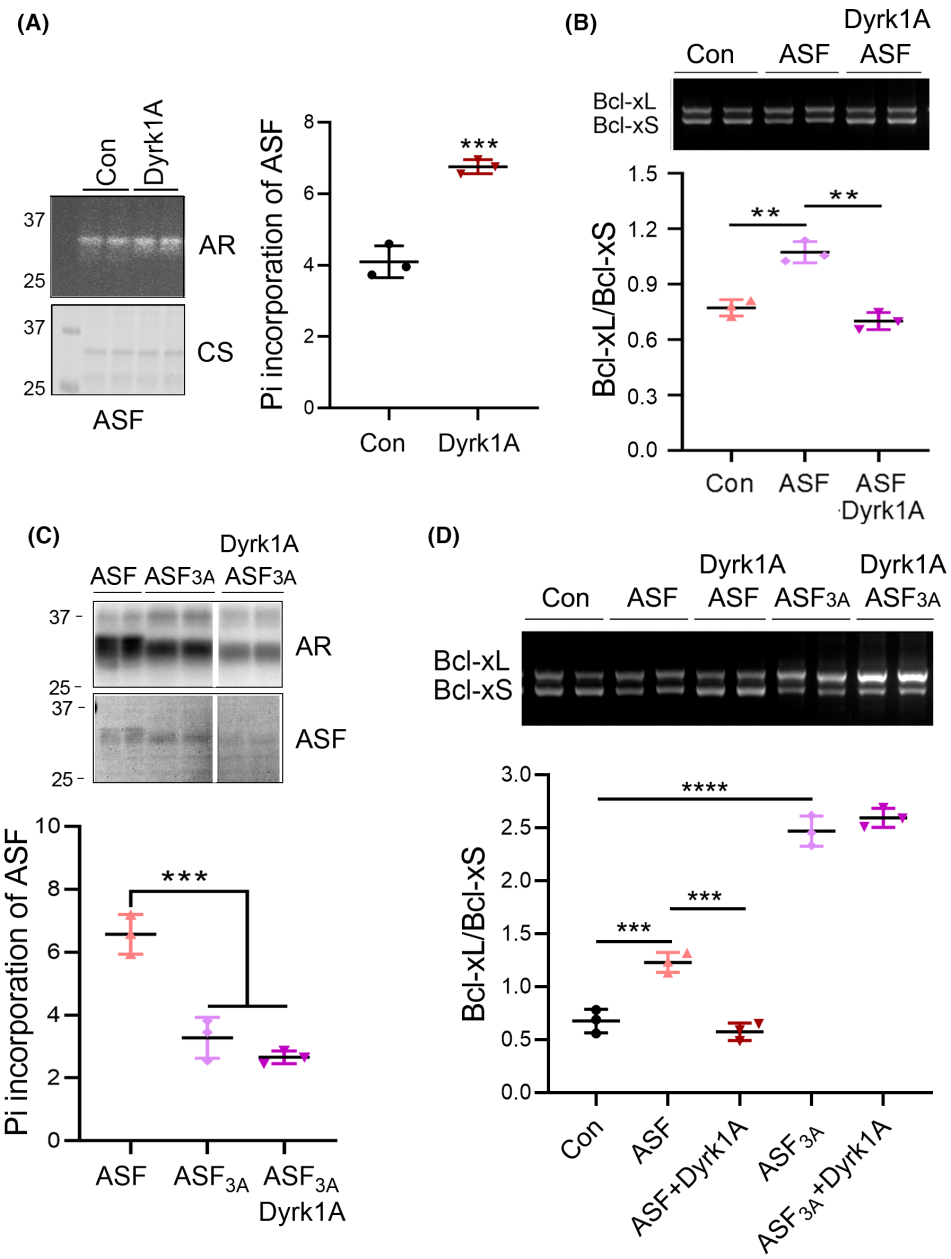
Suppression of ASF‐mediated Bcl‐x exon 2b inclusion by Dyrk1A requires Ser227, Ser234, and Ser238 phosphorylation. (A) HEK‐293FT cells were transfected with Dyrk1A for 45 h and labeled with ^32^Pi for 3 h. The cells were lysed with RIPA buffer. The ASF was immunoprecipitated and separated by SDS‐PAGE. Incorporation of ^32^Pi into ASF was analyzed by autoradiography and normalized by total ASF showed by Coomassie Blue Staining. (B) Dyrk1A and ASF were co‐expressed with Bcl‐x mini‐gene in U87 cells. The splicing products of Bcl‐x exon 2b were analyzed by RT‐PCR. (C) Dyrk1A and mutated ASF, ASF_3A_, were co‐transfected to HEK‐293 T cells for 45 h, ^32^Pi was added to the culture medium for another 3 h. The ASF was immunoprecipitated and separated by SDS‐PAGE. ^32^Pi incorporation was analyzed by autoradiography and normalized with total ASF showed by Coomassie Blue Staining. (D) Dyrk1A and ASF or ASF_3A_ were co‐transfected with Bcl‐x mini‐gene in HEK‐293A cells. The splicing products of Bcl‐x exon 2b were analyzed by RT‐PCR. The Bcl‐xL/Bcl‐xS ratio was calculated. CS, Coomassie Blue Staining; AR, autoradiography; ASF_3A_, Ser227, Ser234, and Ser238 of ASF mutated to Ala. *, compared with Con; #, compared with ASF. Data are presented as mean ± SD, *n* = 3; *, *p* < 0.05; **, *p* < 0.01.

To further explore the role of Dyrk1A on ASF phosphorylation, we overexpressed Dyrk1A in HEK‐293FT cells and labeled cells with [^32^P] orthophosphate. Then, ASF was immunoprecipitated with anti‐ASF and the ^32^Pi incorporation by ASF was analyzed using autoradiography. Overexpression of Dyrk1A significantly increased the ^32^Pi incorporation of ASF (Figure 4A). ASF‐only enhanced Bcl‐xL expression, however, co‐expression with Dyrk1A inhibited ASF‐mediated Bcl‐xL expression in U87 cells (Figure 4B). These results suggest that Dyrk1A phosphorylates and suppresses ASF‐mediated Bcl‐x exon 2b inclusion.

Dyrk1A phosphorylates ASF at Ser227, Ser234, and Ser238.^25^ To study whether the suppression of ASF‐mediated Bcl‐x exon 2b inclusion by Dyrk1A is dependent on the phosphorylation of these three Ser amino acids, we first mutated Ser227, Ser234, and Ser238 to Ala (named as ASF_3A_ here), and overexpressed ASF and ASF_3A_ with or without Dyrk1A in HEK‐293A cells and labeled cells with [^32^P]orthophosphate. Compared with that of wild‐type ASF, the phosphorylation level of ASF_3A_ was decreased, although it did not show a significant difference (Figure 4C). However, co‐expression of Dyrk1A with ASF_3A_ could not enhance ASF_3A_ phosphorylation (Figure 4C). Both ASF and ASF_3A_ could increase Bcl‐xL expression (Figure 4D). However, unlike wild‐type ASF, Dyrk1A failed to inhibit ASF_3A_‐mediated Bcl‐x exon 2b inclusion (Figure 4D). Thus, Dyrk1A suppresses the function of ASF in promoting Bcl‐xL expression through the phosphorylation of Ser227, Ser234, and Ser238.

### 3.5 Deletion of C‐terminus of Dyrk1A exhibited a higher kinase activity and stronger interaction with ASF

Dyrk1A was truncated at the C‐terminus and N‐terminus by over‐activated calpain Ι in vitro and in AD brain, and the truncation increased its kinase activity.^22^ To further study the effect of truncated Dyrk1A on Bcl‐x exon 2b expression, we constructed a series of Dyrk1A deletion mutants, progressively deleted from the C‐terminus and N‐terminus, named as Dyrk1A_1–673_, Dyrk1A_1–625_, Dyrk1A_1–597_, Dyrk1A_1–531_, Dyrk1A_1–483_, Dyrk1A_100–763_, Dyrk1A_156–763_, and Dyrk1A_156–483_ (Figure S1A). These plasmids were tagged with FLAG at the C‐terminus. The expression levels of full length Dyrk1A and truncated Dyrk1A were quite similar (Figure S1B). Dyrk1A contains two nuclear localization signals (NLS) with one near the N‐terminus of the kinase domain and the other within the kinase domain. The poly‐histidine domain (His) at the C‐terminus of Dyrk1A is critical for its nuclear speckle localization, where Dyrk1A phosphorylates splicing factors and keeps them inactive. Dyrk1A_1–763_, Dyrk1A_1–673_, Dyrk1A_1–625_, and Dyrk1A_100–763_ with the His domain were localized in the nuclear speckles (Figure S1C). For mutants without the His domain, Dyrk1A_1–597_, Dyrk1A_1–531_, Dyrk1A_1–483_, and Dyrk1A_156–483_ were evenly distributed in the nucleus (Figure S1C). Dyrk1A_156–763_ and Dyrk1A_156–483_ without the first NLS domain were mainly localized at nucleus and only a small partial Dyrk1A translocated to cytoplasm (Figure S1C), indicating that the second NLS domain is important for the nuclear localization of Dyrk1A. However, Dyrk1A_156–763_ with the His domain could not localize at the nuclear speckles (Figure S1C). The exact mechanism remains unknown. Some domains within the region from a.a.101 to 155 may affect its localization at nuclear speckles. These data indicate that the truncated Dyrk1A without the His domain could not localize at the nuclear speckles.

**FIGURE 5:**
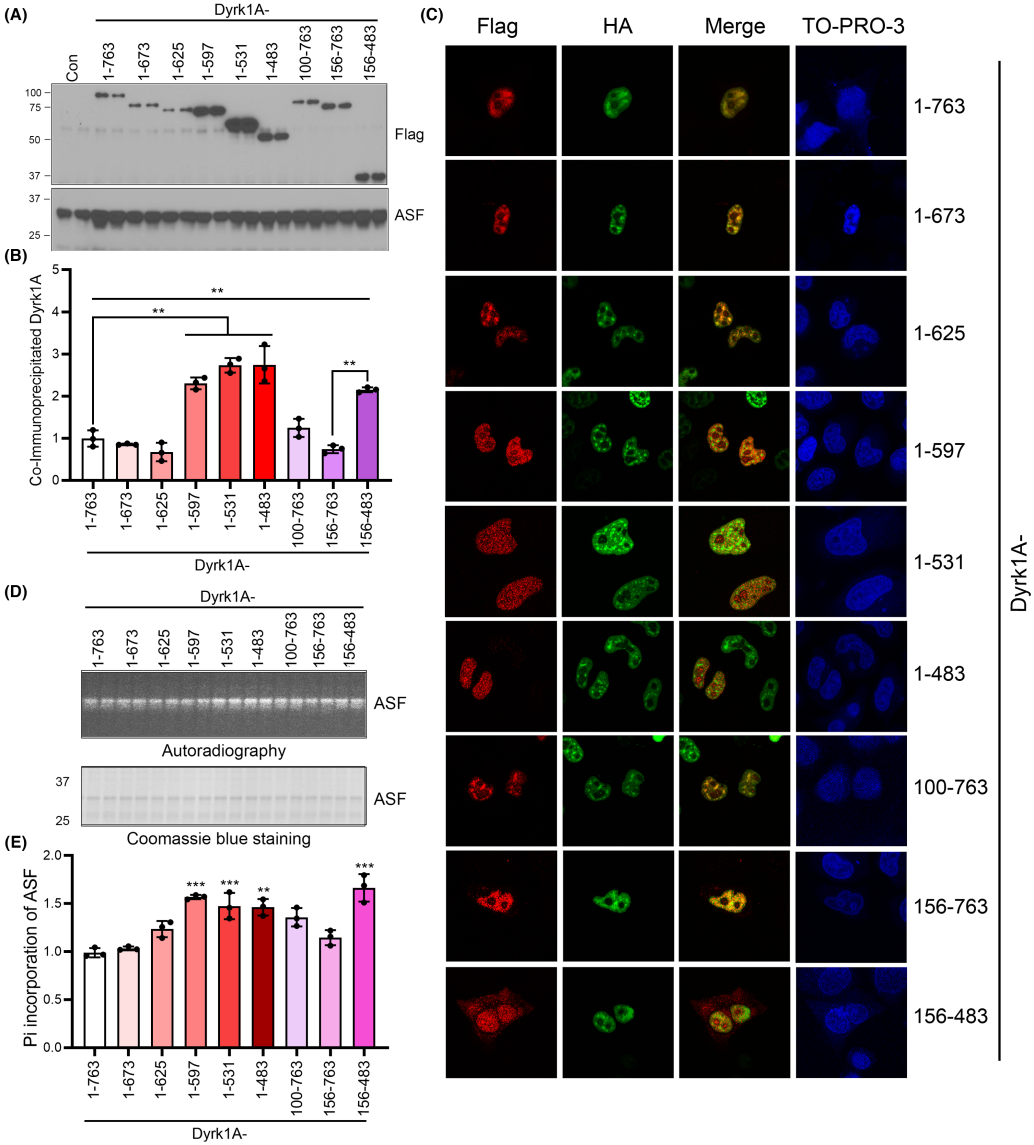
Truncated Dyrk1As without the C‐terminus enhanced its interaction with ASF and the phosphorylation level of ASF. (A,B) Dyrk1A and its series deletions were transfected to HEK‐293FT cells for 48 h. The cells were lysed and incubated with ASF antibody to immunoprecipitate endogenous ASF. The co‐immunoprecipitated Dyrk1A was detected by western blotting using anti‐FLAG antibody (A). The co‐immunoprecipitated Dyrk1A was normalized with total Dyrk1A (showed in Figure S1B) (B). (C) Dyrk1A or Dyrk1A truncation mutants and ASF were co‐transfected into Hela cells. Polyclonal anti‐FLAG and monoclonal anti‐HA were used to immunostain Dyrk1A and ASF respectively. The fluorescent‐labeled anti‐mouse (green) and anti‐rabbit (red) antibodies were used to check the overexpressed protein. TO‐PRO‐3 was used for nuclear staining. (D,E) The HEK‐293FT cells were transfected with Dyrk1A and its deletion mutants and treated with ^32^Pi medium for 3 h. The cells were lysed with RIPA buffer. The monoclonal ASF antibody was used to immunoprecipitate endogenous ASF. The phosphorylation levels of ASF were checked by autoradiography (D) and normalized with total ASF (showed by Coomassie Blue Staining) (E). CS, Coomassie Blue Staining; AR, autoradiography; Data are presented as mean ± SD, *n* = 3; *, *p* < 0.05; **, *p* < 0.01.

To further investigate the effect of truncated Dyrk1A on its ability to interact and phosphorylate ASF, we transfected Dyrk1A and its deletions to HEK‐293FT cells (Figure 5A,B) or with HA‐tagged ASF to HeLa cells (Figure 5C). Compared with that of Dyrk1A_1–763_, the level of co‐immunoprecipitated Dyrk1A_1–597_, Dyrk1A_1–531_, and Dyrk1A_1–483_ by endogenous ASF increased drastically (>2‐folds), but not the Dyrk1A_1–673_ and Dyrk1A_1–625_ (Figure 5A,B). The truncated Dyrk1A, without 598–626 a.a. at the C‐terminus including His domain, accelerated their interaction with ASF, indicating that this domain may hinder the interaction of Dyrk1A with ASF. The level of co‐immunoprecipitated Dyrk1A_100–763_ and Dyrk1A_156–763_ by ASF were similar to that of full length Dyrk1A (Figure 5A,B). Deleting the first 1–155 a.a. at the N‐terminus of Dyrk1A did not affect its interaction with ASF (Figure 5A,B). Compared with Dyrk1A_156–763_, much more Dyrk1A_156–483_, deleted at the C‐terminus, was co‐immunoprecipitated by ASF, confirming that the C‐terminus of Dyrk1A, not the N‐terminus, could inhibit its interaction with ASF. Dyrk1A_1–763_, Dyrk1A_1–673_, Dyrk1A_1–625_, and Dyrk1A_100–763_ co‐localized with ASF at the nuclear speckles (Figure 5C). However, Dyrk1A_1–597_, Dyrk1A_1–531_, Dyrk1A_1–483_, Dyrk1A_156–763_, and Dyrk1A_156–483_ were evenly distributed in the nucleus and had no effect on the nuclear speckle localization of ASF (Figure 5C). Consistent with co‐immunoprecipitation results, the phosphorylation level of ASF significantly increased when the cells were transfected with Dyrk1A_1–597_, Dyrk1A_1–531_, Dyrk1A_1–483_, and Dyrk1A_156–483_, but not with the Dyrk1A_1–673_, Dyrk1A_1–625_, Dyrk1A_100–763_, and Dyrk1A_156–763_ (Figure 5D). These evidences indicate that the truncated Dyrk1A without C‐terminus promoted its interaction with ASF and ASF phosphorylation.

### 3.6 Increased Bcl‐xS expression and significant enrichment of apoptosis‐related genes may be caused by the truncated Dyrk1A in 5xFAD mice

**FIGURE 6:**
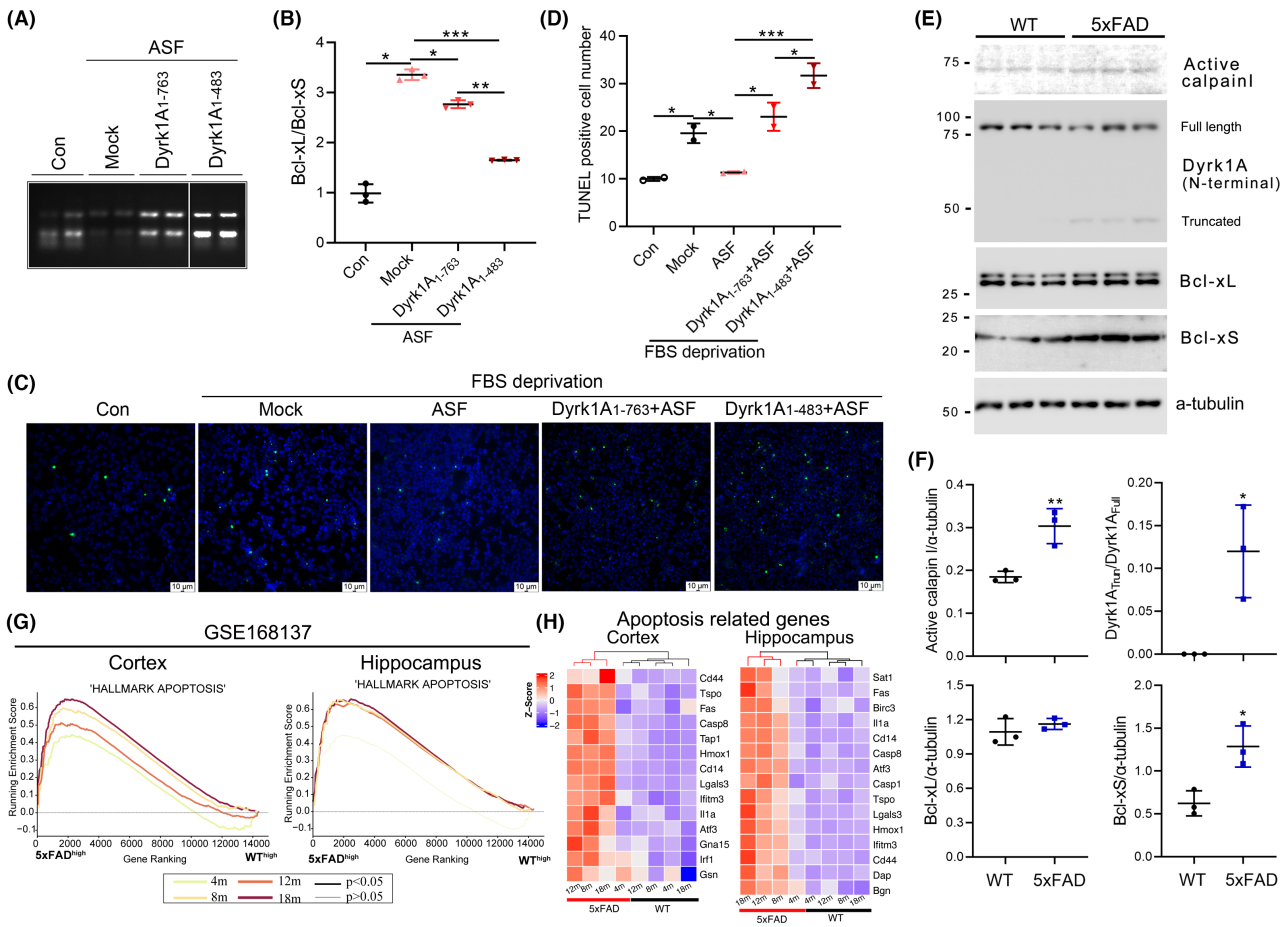
Truncation of Dyrk1A may cause decreased Bcl‐xL and increased apoptosis gene enrichment in 5xFAD mice. (A,B) Dyrk1A_1–763_ or Dyrk1A_1–483_ and ASF were co‐transfected to HEK‐293 T cells. The total RNA was extracted to check the expression of Bcl‐xL and Bcl‐xS (A). The Bcl‐xL/Bcl‐xS ratio was calculated (B). (C,D) HEK‐293 T cells were co‐transfected with ASF, Dyrk1A_1–763_, or Dyrk1A_1–483_ for 24 h. The cells were deprived of FBS to induce apoptosis and were then subjected TUNEL staining (C). The TUNEL positive cells were counted in every 1000 cells (D). (E,F) The protein level of activated calpain I, full length and truncated Dyrk1A, Bcl‐xL, and Bcl‐xS in 5xFAD cortex at 10 m were checked by western blotting using calpain I, Dyrk1A (N‐terminal), Bcl‐xL, and Bcl‐xS antibodies (E). The densities of activated calpain I, Bcl‐xL, and Bcl‐xS bands were normalized to a‐tubulin, and the truncated Dyrk1A was normalized to the full length Dyrk1A (F). (G) Apoptosis gene enrichment was analyzed by GESA from hippocampal (right) and cortex (left) RNA sequencing data of 5xFAD mice at 4 m, 8 m, 12 m, and 18 m (GSE168137). (H) Apoptosis‐related 14 or 15 gene expression patterns were increased in 5xFAD mice, as represented by *Z* scores in heatmap. Thin curve, *p* > 0.05, thick curve, *p* < 0.05. Data are presented as mean ± SD, for cell experiment *n* = 3, for animal experiment *n* = 2; *, *p* < 0.05; **, *p* < 0.01; ***, *p* < 0.001.

The truncated Dyrk1A without the C‐terminus, not the N‐terminus, enhanced its interaction with and kinase activity toward ASF. To examine the effect of truncated Dyrk1A on Bcl‐x expression and apoptosis, we co‐overexpressed Dyrk1A_1–763_ and Dyrk1A_1–483_ with ASF in Bcl‐x‐transfected HEK‐293FT cells. ASF could promote the inclusion of Bcl‐x exon 2b, whereas co‐expression of Dyrk1A_1–763_ or Dyrk1A_1–483_ with ASF suppressed the ASF‐mediated enhancement of Bcl‐x exon 2b inclusion. However, the biological activity of Dyrk1A_1–483_ in suppressing the function of ASF was much stronger than that of full length Dyrk1A_1–763_ (Figure 6A,B).

Further, we analyzed the effect of Dyrk1A_1–763_ and Dyrk1A_1–483_ on apoptosis by TUNEL assay (Figure 6C). Compared with the control group (Con), FBS deprivation (Mock) increased TUNEL‐positive cell number. However, ASF drastically decreased the TNUEL‐positive cell number caused by FBS deprivation. Under FBS deprivation, co‐expression of Dyrk1A_1–763_ and Dyrk1A_1–483_ with ASF both enhanced the number of apoptotic cells, which was more apparent in Dyrk1A_1–483_ group. (Figure 6C,D).

Aβ can disrupt the Ca^2+^ homeostasis and induce the activation of protease calpain I.^34^, ^35^ However, the effect of Aβ on the truncation of Dyrk1A by activating calpain I remains unknown. Therefore, we checked the Dyrk1A in the cortex of AD model 5xFAD mice at 10 months (m). Aβ and the increased activated calpain was observed in 5xFAD mice (Figure 6E,F). The Dyrk1A antibody, against the N‐terminal of Dyrk1A, was used to check the full length and truncated Dyrk1A. The truncated Dyrk1A under 50‐kDa only existed in 5xFAD mice, not in the wild‐type mice (Figure 6E,F), indicating that the truncated Dyrk1A is quite close to Dyrk1A_1–483_ by molecular weight. Consistent with the in vitro result, a significant increase in Bcl‐xS was observed in 5xFAD mice while the expression of Bcl‐xL remained unaltered. (Figure 6E,F). To confirm the increased apoptosis in AD brain, we reanalyzed RNA sequencing dataset of cortex and hippocampus in the 5xFAD mice at 4 m, 8 m, 12 m, and 18 m (GSE168137).^36^ The apoptosis‐related genes were significantly enriched in the cortex at 4 m, 8 m, 12 m, and 18 m, and the hippocampus at 8 m, 12 m, and 18 m, but not at 4 m in AD mice, compared with those in the corresponding tissues of the wild‐type mice (Figure 6G), indicating the enhanced apoptosis in AD mice. Of the apoptosis‐associated genes, we noted genes, such as 18 kDa translocator protein (TSPO),^37^, ^38^ *heme oxygenase 1 (Hmox1),* ^39^ caspase 8 (Casp8),^40^ that were significantly increased in 5xFAD mice (Figure 6H). Thus, compared with full length Dyrk1A, the truncated Dyrk1A_1–483_ had a higher biological activity to promote neuronal apoptosis by inhibiting the expression of anti‐apoptotic Bcl‐xL in AD.

## 4 DISCUSSION

*Dyrk1A* gene is located at DS critical region. Dyrk1A plays vital roles in the onset and development of AD in patients with DS, through Aβ and tau pathology.^12^, ^22^ However, the role and the underlying mechanism of Dyrk1A in the neuronal loss in AD is less investigated. In the present study, we found that Dyrk1A overexpression in wild‐type mice causes increased expression of apoptosis‐related genes in the hippocampus. Overexpression of Dyrk1A enhanced Bcl‐xS expression and apoptosis in cultured cells. We also found that ASF enhanced the expression of Bcl‐xL promoting cell survival. However, Dyrk1A phosphorylated ASF and suppressed ASF‐mediated Bcl‐xL expression via the phosphorylation of Ser227, −234, and − 238 residues on ASF. Moreover, the C‐terminus, and not the N‐terminus, truncated Dyrk1A, translocated from the nuclear speckle to the whole nucleoplasm, and enhanced its affinity and kinase activity toward ASF, leading to decreased Bcl‐xL/Bcl‐xS ratio and aggravated cell apoptosis. Therefore, Dyrk1A may serve as a pro‐apoptotic protein via promotion of Bcl‐xS expression, and the C‐terminus‐truncated Dyrk1A further enhanced its pro‐apoptotic function.

Bcl‐x, a Bcl2 family protein, located at the outer mitochondrial membrane, regulates cell survival and death. In the CNS, Bcl‐x splicing plays a vital role in modulating neuronal apoptosis in both developing and mature CNS. The apoptotic stimuli could reduce Bcl‐xL and increase Bcl‐xS levels, suggesting that the alternative splicing of Bcl‐x may influence the neuronal apoptosis.^41^ Knockdown of Bcl‐xS in the rat hippocampus reduced the neuronal vulnerability to the injury induced by hypoxia–ischemia.^41^ Bcl‐xL could promote neuronal mitochondrial function by interacting with mitochondrial F_1_F_o_ ATP synthase,^42^ inhibiting PINK1/Parkin‐dependent mitophagy^43^ and increasing mitochondrial biomass.^44^ The transplantation of TAT‐Bcl‐xL‐neural precursor cells and neuronal overexpression of Bcl‐xL resulted in long‐term neuroprotection and ameliorated the functional deficits after cerebral ischemia in mice.^45^ Thus, the imbalance of Bcl‐xL and Bcl‐xS may induce neuronal dysfunction and apoptosis. Here, we found that Dyrk1A could promote apoptosis by reducing Bcl‐xL and increasing Bcl‐xS, leading to neuronal apoptosis in AD.

Dyrk1A regulates alternative splicing via phosphorylation of splicing factors (SRs) and affects their function.^25^, ^27^, ^28^ ASF is a well‐studied splicing factor and upregulated in various human tumors. ASF transgenic mice develop cancer in multiple organs.^46^ We have previously reported that Dyrk1A phosphorylates ASF and suppresses its function in tau exon 10 inclusion.^25^ Here, Dyrk1A phosphorylated ASF and suppressed ASF‐mediated Bcl‐xL expression.

Dyrk1A is overexpressed in DS due to the trisomy. Increased apoptosis in the brains of individuals with DS is believed to contribute to mental retardation and early onset neurodegeneration.^21^ In addition, prevalence of solid tumors is extremely low in DS.^47^, ^48^ Recently, Pozo et al., based on their experiments involving the pharmacological inhibition of Dyrk1A in tumor‐initiating cells and tumor specimens, suggested that Dyrk1A could represent a promising therapeutic target in EGFR‐dependent glioblastoma.^49^ Notably, Dyrk1A is recognized as both tumor suppressor and oncogene. Here, our results indicate that Dyrk1A inhibited the inclusion of Bcl‐x exon 2b, leading to the increase in pro‐apoptotic Bcl‐xS expression, suggesting that Dyrk1A may serve as a pro‐apoptotic protein to promote neuronal apoptosis in DS and AD.

A widely used AD mouse model, 5xFAD, expresses five familial AD mutations and is characterized by the Aβ plaques. Accumulating evidence indicates that Aβ can disrupt the cellular Ca^2+^ homeostasis.^34^, ^35^ Aβ increased the calpain I activity in primary neuronal cultures^50^ and transgenic mice with human APP mutation.^34^, ^51^ Therefore, we speculate that Aβ could cause Dyrk1A truncation that aggravated apoptosis in 5xFAD mice. Consistent with this hypothesis, truncated Dyrk1A and significant increase in the expression of apoptosis‐related genes were observed in the brain of 5xFAD mice (Figure 6E,G). Our findings are also consistent with the increased apoptosis observed in the brains of adult DS individuals with AD pathology.^52^, ^53^

The overactivated calpain Ι proteolyzes Dyrk1A in vitro and in AD frontal cortices. The proteolyzed Dyrk1A without the C‐terminal could promote 3R‐tau expression and tau hyperphosphorylation, contributing to tau pathology in AD.^22^ The proteolyzed Dyrk1A in the hippocampus of patients with AD has stronger affinity toward the inflammatory regulator STAT3a. Intraperitoneal injection of Leucettine L41, a compound preventing Dyrk1A proteolysis, inhibits STAT3a phosphorylation and decreases pro‐inflammatory cytokine expression levels (IL1‐β, TNF‐α, and IL‐12) and ameliorates amyloid plaque load, synaptic plasticity, and cognitive functions in aged APP/PS1 mice.^54^ Except for the crucial role of truncated Dyrk1A in AD pathology, autism‐associated truncation mutations of Dyrk1A, R205X, and E239X, cause loss of function and lead to defects in the dendritic outgrowth, dendritic spine density, and cortical migration during neuron development in autism spectrum disorder.^55^ The gain or loss of function of Dyrk1A due to abnormal truncation can affect neuronal development or neurodegeneration in neurological disorders. In this study, we demonstrated that the truncated Dyrk1A without the histidine repeat and serine/threonine‐rich domain, Dyrk1A_1–597_, Dyrk1A_1–531_, Dyrk1A_1–483_, and Dyrk1A_156–483_, enhanced their affinity and kinase activity toward ASF to inhibit ASF‐mediated Bcl‐x exon 2b inclusion. Compared with that of wild‐type Dyrk1A, the decreased ratio of Bcl‐xL/Bcl‐xS and apoptosis were further increased by the co‐expression of Dyrk1A_1–483_ in HEK‐293 T cells. Thus, the proteolyzed Dyrk1A may promote neurodegeneration through apoptosis in AD. The present study, with this newly identified mechanism, will contribute to the development of novel therapeutic approach in AD.

## AUTHOR CONTRIBUTIONS

S.Z., J.Z., L.X., Y.W., and N.J. performed the study. G.Z., J.X., and J.S. provided the suggestions on the study designs, and contributed to the manuscript. X.L. and N.J. designed the study and wrote the manuscript. All authors approved the final manuscript.

## CONFLICT OF INTEREST STATEMENT

The authors declare that they have no competing interests.

## Supporting information


Data S1.

Figure S1.

## Data Availability

The expression profiles of hippocampus in the Dyrk1A overexpression mice (TgDyrk1A) (GSE149464) or hippocampus and cortex in the AD model 5xFAD mice (GSE168137) were retrieved from the NCBI GEO database. Other additional information generated and/or analyzed in this paper is available from the corresponding author on reasonable request.
