# Supplementary material for: Truncated Dyrk1A aggravates neuronal apoptosis by inhibiting ASF‐mediated Bcl‐x exon 2b inclusion
Source: CNS Neurosci Ther. 2023 Oct 21;30(4):e14493. doi: 10.1111/cns.14493 (PMC11017436; doi:10.1111/cns.14493)

Full unedited blot for Figure 1F

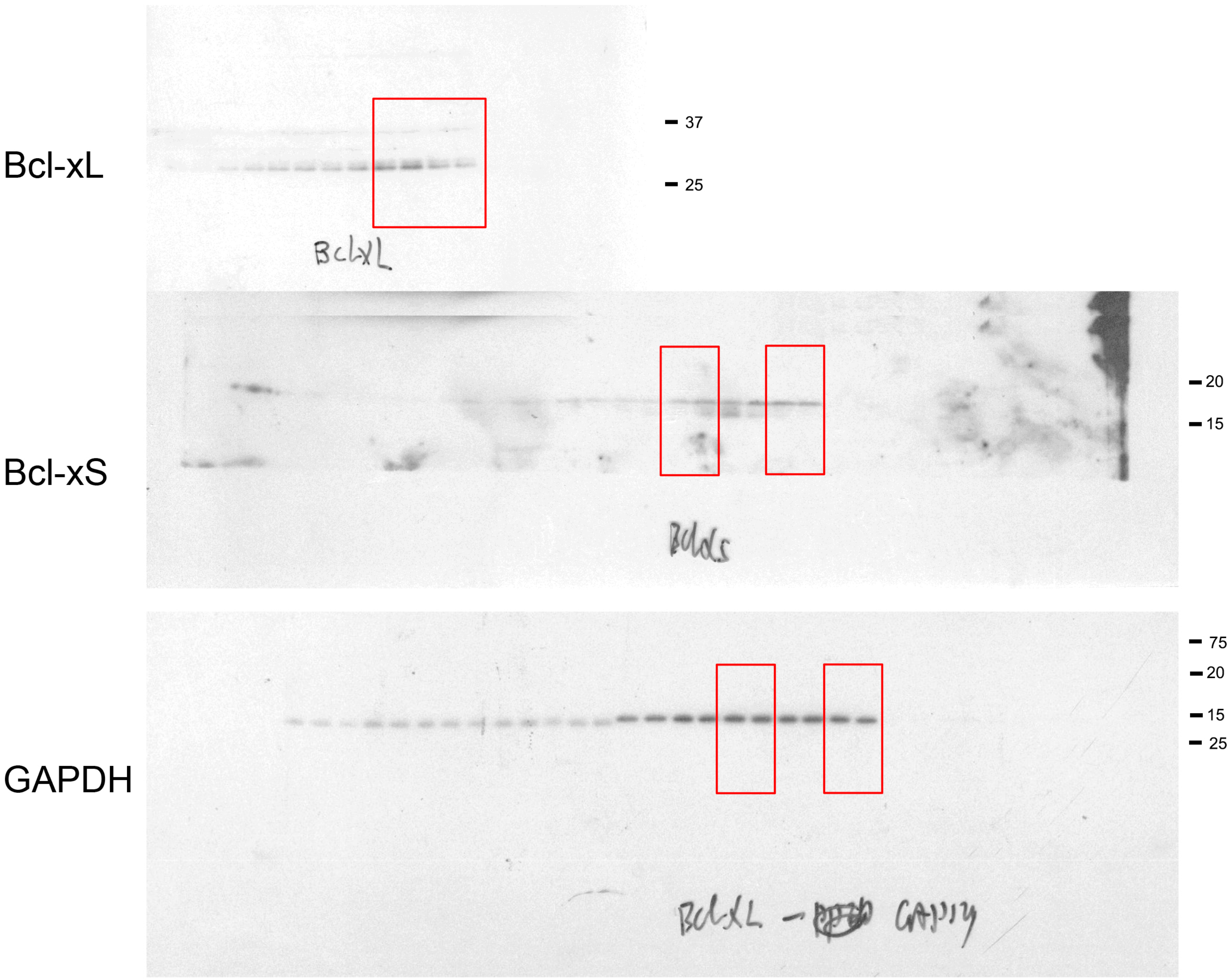

Full unedited blot for Figure 2D

Dyrk1A

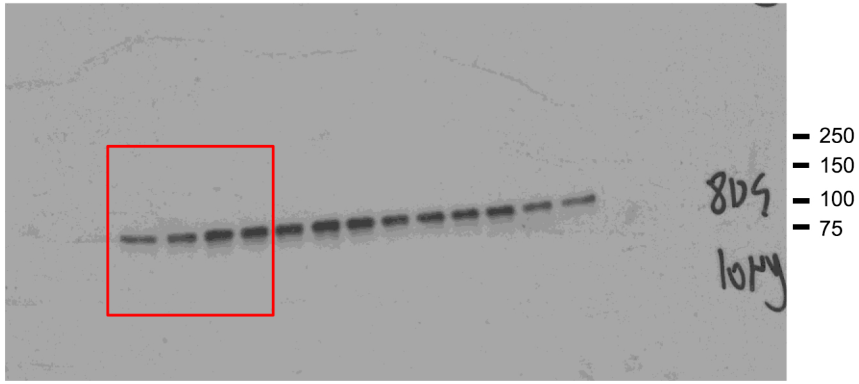

GAPDH

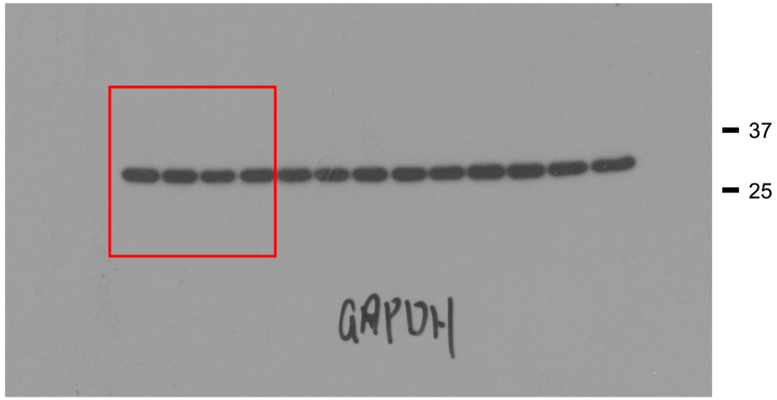

Dyrk1A

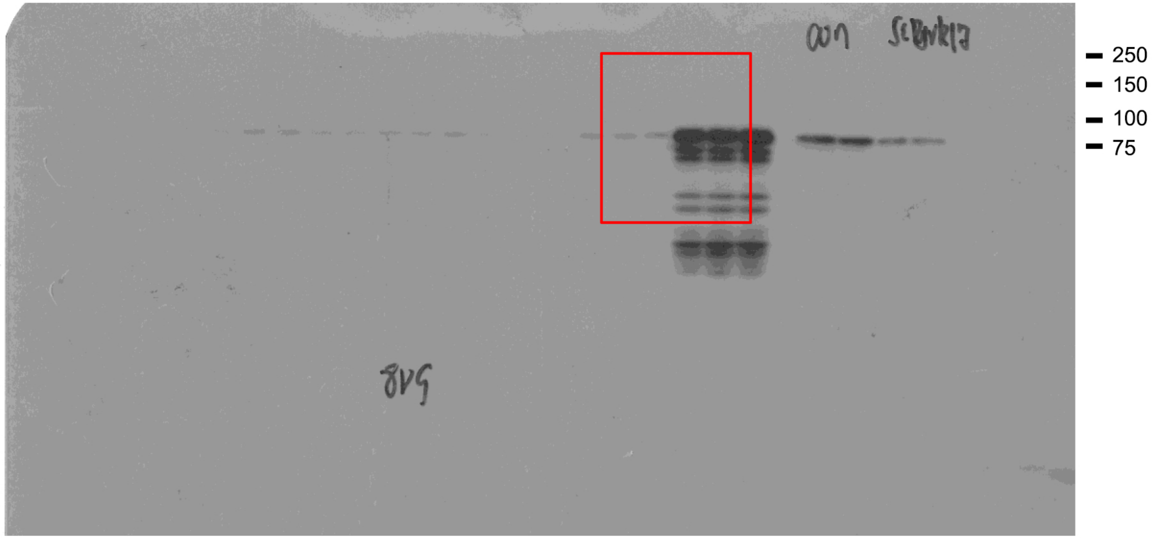

GAPDH

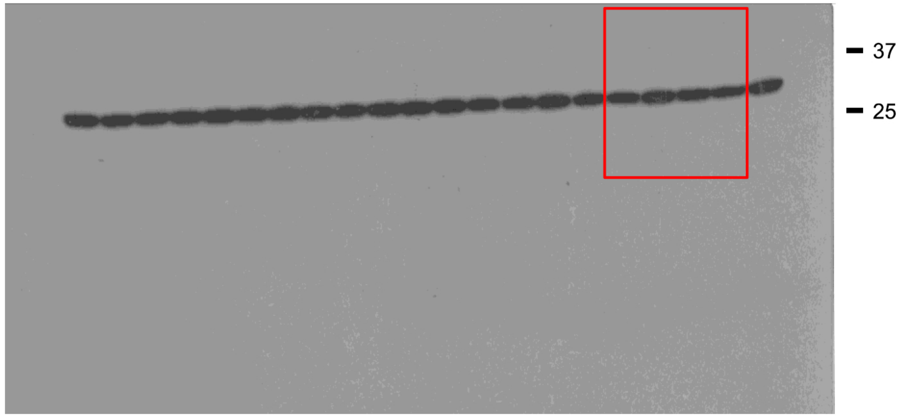

Full unedited blot for Figure 3E

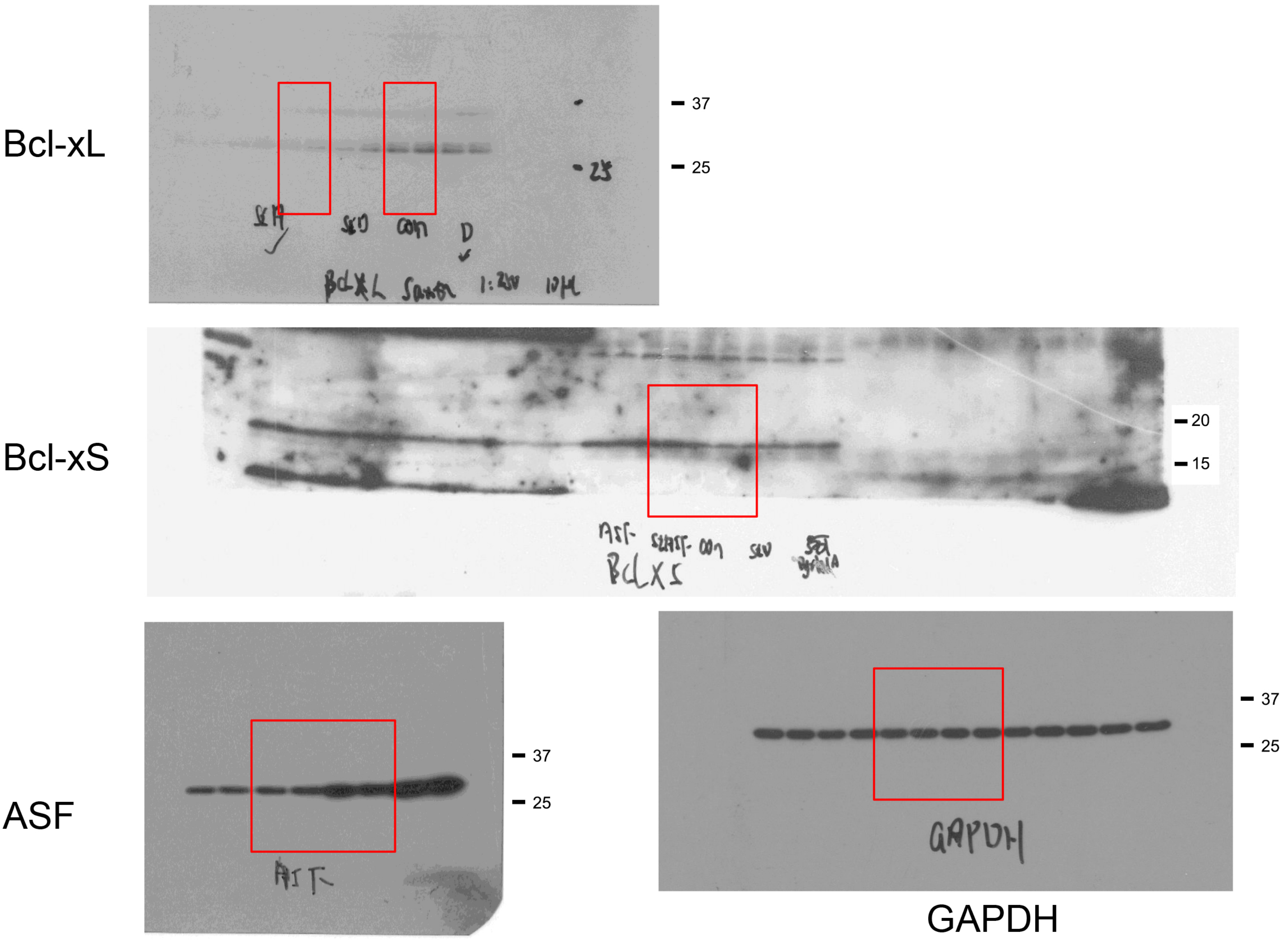

Full unedited blot for Figure 4A

ASF

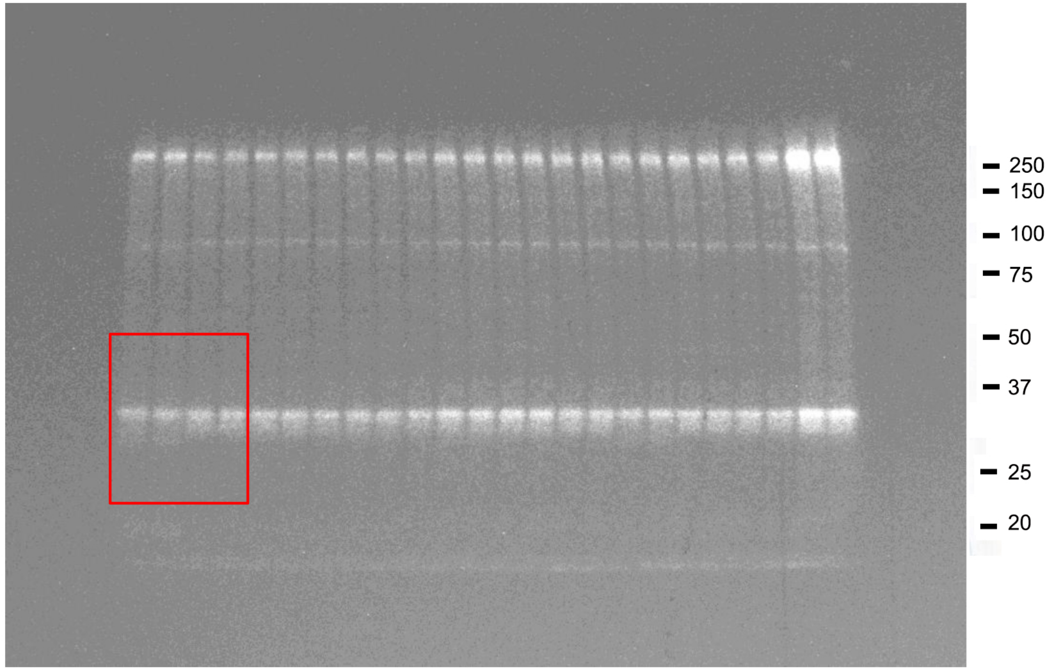

ASF

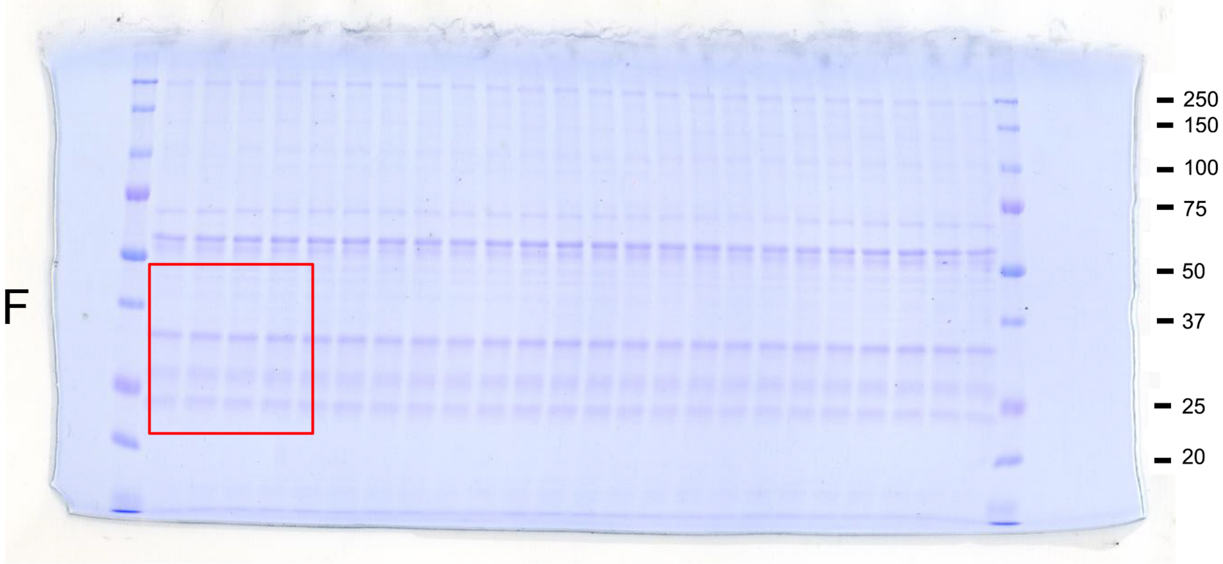

Full unedited blot for Figure 4C

ASF

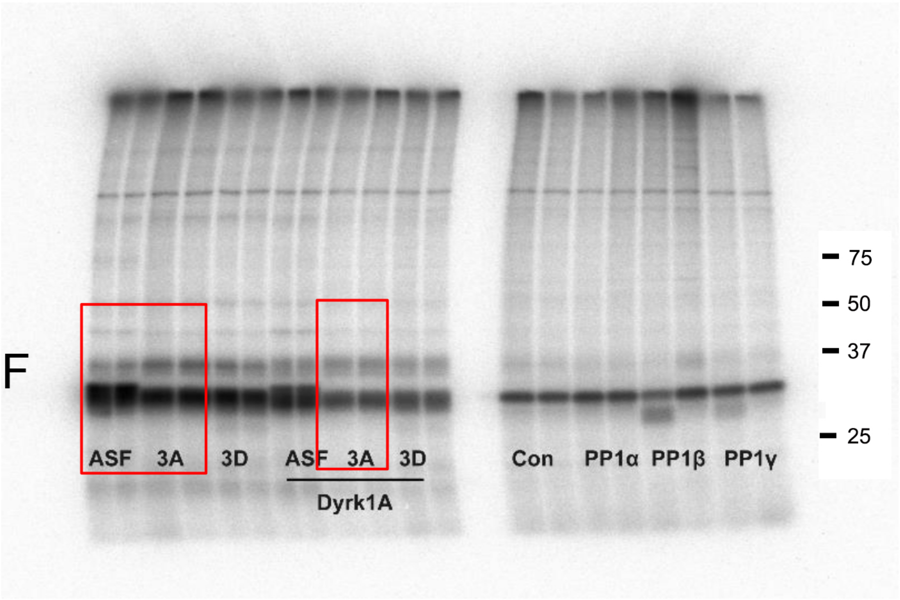

ASF

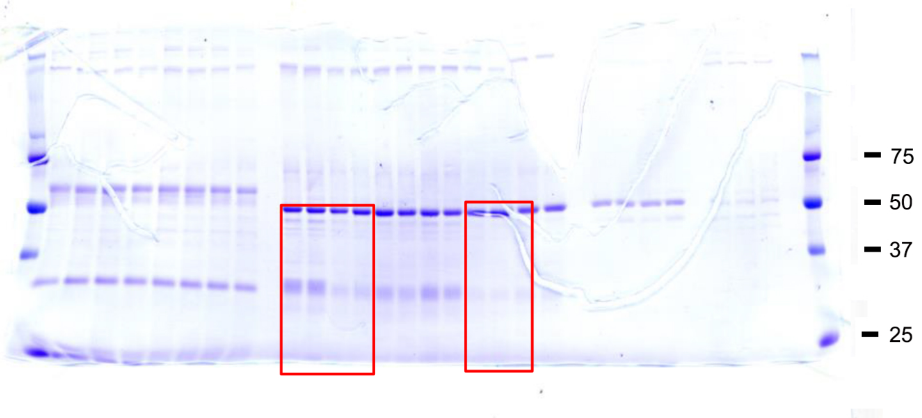

Full unedited blot for Figure 5A

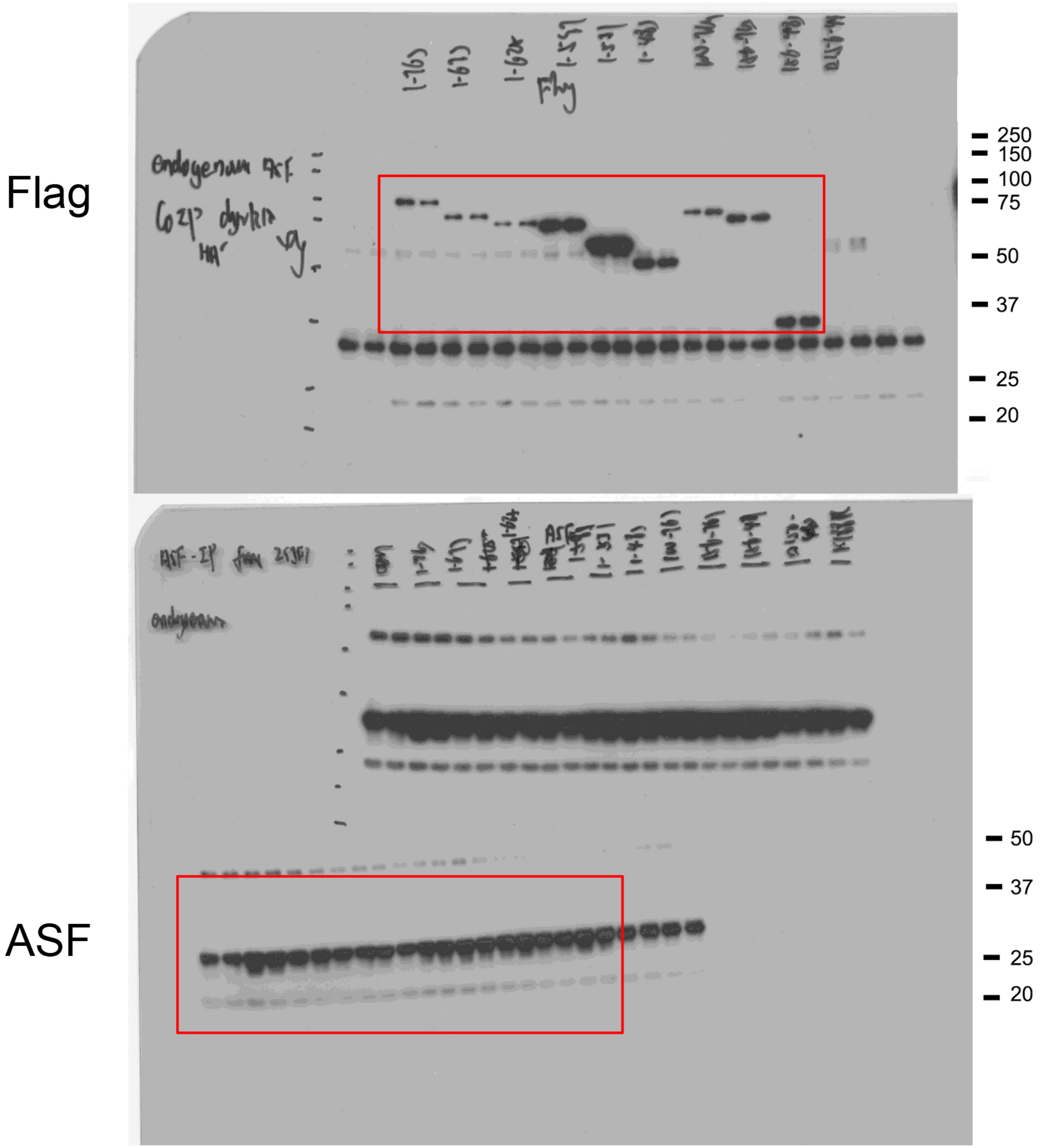

Full unedited blot for Figure 5D

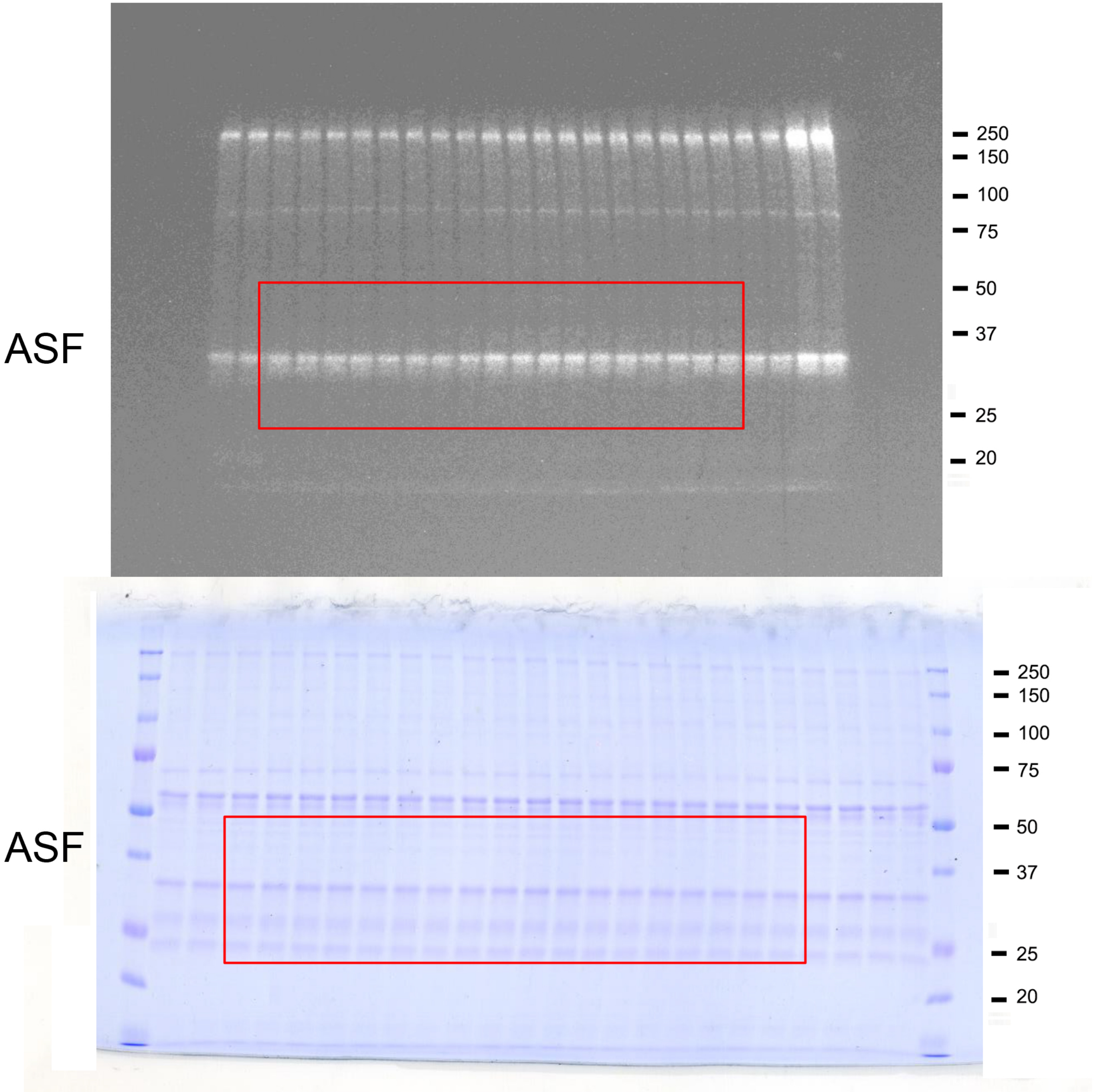

Full unedited blot for Figure 6E

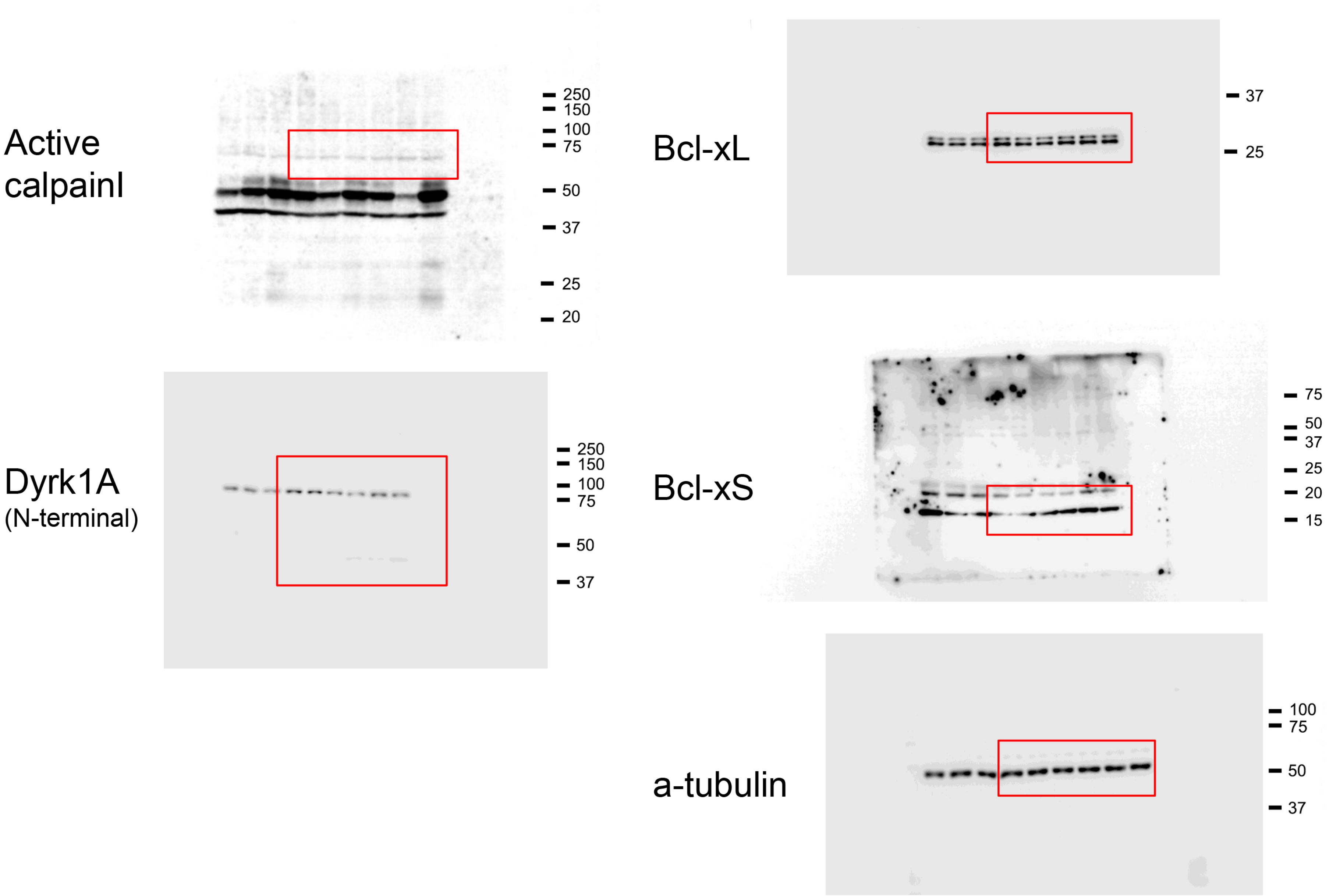

Full unedited blot for Figure S1B

Flag

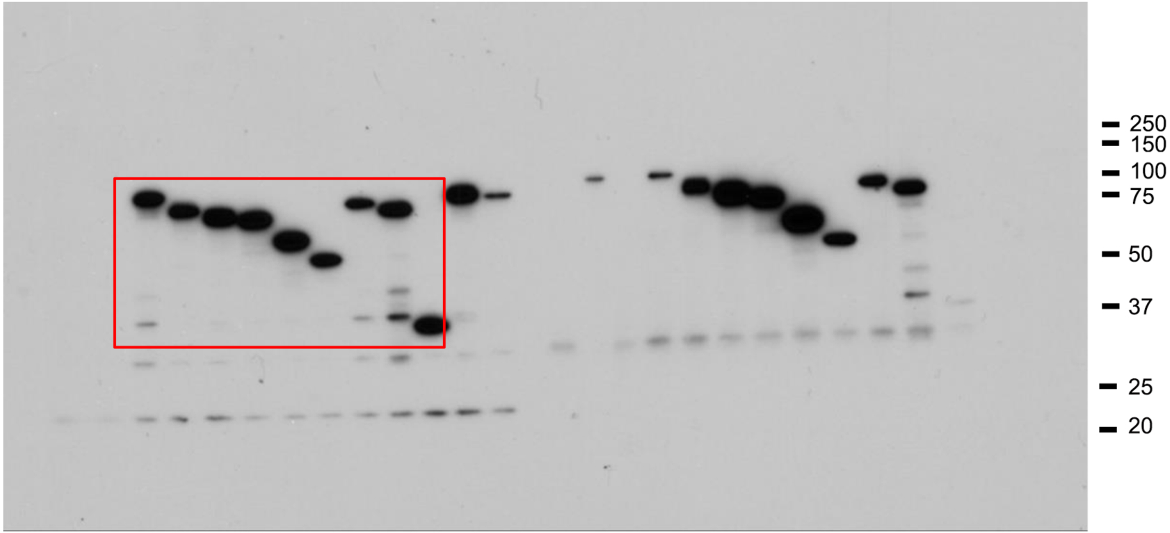

Supplement: Supplementary file 1 — Data S1. [file CNS-30-e14493-s001.pdf]
