# Supplementary material for: Truncated Dyrk1A aggravates neuronal apoptosis by inhibiting ASF‐mediated Bcl‐x exon 2b inclusion
Source: CNS Neurosci Ther. 2023 Oct 21;30(4):e14493. doi: 10.1111/cns.14493 (PMC11017436; doi:10.1111/cns.14493)
Supplement: Supplementary file 2 — Figure S1. [file CNS-30-e14493-s002.docx]

**Supplementary material for review：**

**
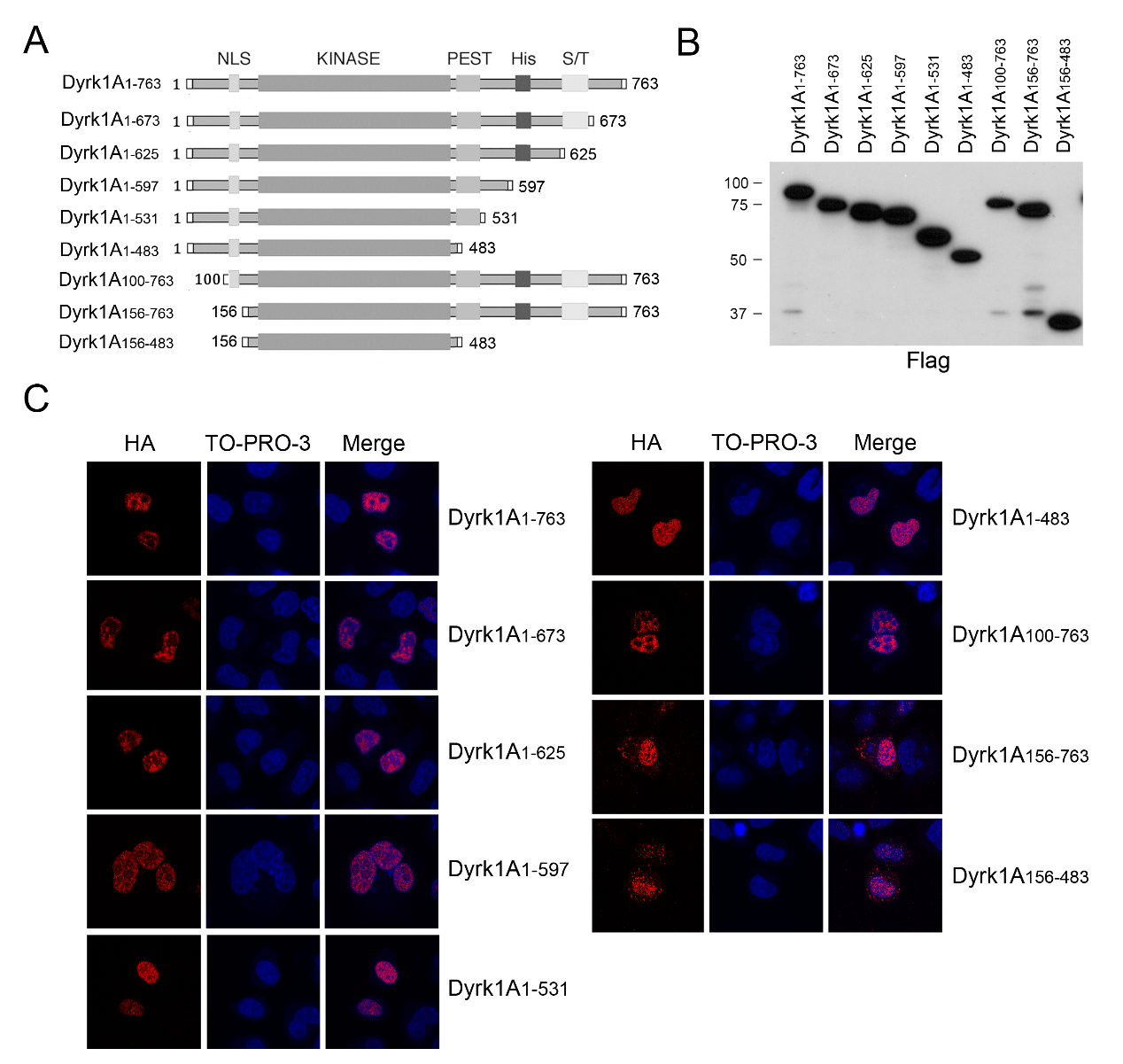
**

**Fig. S1. Histidine repeat domain is required for Dyrk1A to localize at nuclear speckles**. (**A**) Schematic representation of Dyrk1A domain structures and deletion mutants. (**B**) Dyrk1A and its deletion mutants were transfected to HEK-293FT cells. The expression of Dyrk1A was checked by Western blots with FLAG antibody. (**C**) The Hela cells were transfected with Dyrk1A and its deletion mutants followed by immunostaining using polyclonal FLAG antibody and anti-rabbit fluorescence-labeled secondary antibody (Red) to check the subcellular localization of Dyrk1A. TO-PRO-3 was used for nuclear staining. NLS, nuclear localization signal; PEST, peptide sequence that is rich in proline, glutamic acid, serine, and threonine; His, histidine repeat domain; S/T, serine, and threonine rich domain.
